# ROS/pH‐Responsive Phenylboronic Acid–Chitosan Nanoparticles Improve Silymarin‐Mediated Hepatoprotection After Hepatectomy With Gut‐Liver Axis‐Associated Responses

**DOI:** 10.1002/advs.77847

**Published:** 2026-09-27

**Authors:** Jinjin Tong, Chaochao Luo, Zezheng Liu, Xinyan Zhang, Daixu Jia, Yang Sun, Zhaonan Zhang, Haoran Shi, Jianfang Wang, Hua Zhang

**Affiliations:** ^1^ Animal Science and Technology College Beijing University of Agriculture Beijing People's Republic of China; ^2^ College of Life Sciences Shihezi University Shihezi Xinjiang China; ^3^ College of Veterinary Medicine Beijing University of Agriculture Beijing People's Republic of China; ^4^ National Immunization Program Chinese Center for Disease Control and Prevention Beijing People's Republic of China; ^5^ Department of Biochemical Engineering University College London London UK

**Keywords:** antimicrobial, chitosan, gut–liver axis, hepatectomy, nanoparticles, ROS‐responsive, silymarin

## Abstract

To improve hepatoprotective delivery after hepatectomy, a ROS‐ and pH‐responsive silymarin nanoplatform was constructed using phenylboronic acid‐grafted chitosan (Sil@PBACS Nps). The nanoparticles displayed a uniform size of 250–300 nm, high encapsulation efficiency, and favorable colloidal stability. Their release behavior was accelerated under acidic and ROS‐enriched conditions, supporting lesion‐responsive silymarin delivery. Compared with free silymarin, Sil@PBACS Nps (Nps) exhibited stronger antioxidant activity, with DPPH and ABTS scavenging rates of 92.31% and 73.78%, respectively, together with moderate antibacterial effects against *S. aureus* and *E. coli*. The formulation showed good biocompatibility, with hemolysis below 5% and cell viability above 80% in AML‐12 and HepG2 cells. In a canine hepatectomy model, oral Sil@PBACS Nps reshaped gut microbial composition and altered fecal metabolites associated with sphingolipid metabolism and the pentose phosphate pathway, while hepatic proteomics indicated pathway‐level changes related to oxidative phosphorylation and fatty acid biosynthesis. Further validation of the targeted proteins in the mouse model confirmed the involvement of intestinal barrier‐related responses in the mouse model and hepatic inflammatory, bile acid, antioxidant, lipid‐metabolic, and apoptosis/recovery‐associated pathways in both murine liver tissues. These results suggest that Sil@PBACS Nps facilitate post‐hepatectomy liver recovery by integrating stimuli‐responsive delivery, redox regulation, and gut–liver axis‐associated hepatic protection.

## Graphical abstract

Bioactive phenylboronic acid–chitosan nanoparticles enable responsive silymarin delivery for post‐hepatectomy liver protection. The nanoplatform integrates antioxidant and antibacterial functions, improves hepatic recovery, and is associated with coordinated shifts in gut microbiota, metabolites, cytokines, and hepatic proteomic pathways, suggesting a microbiota–hepatic metabolic link in postoperative repair.

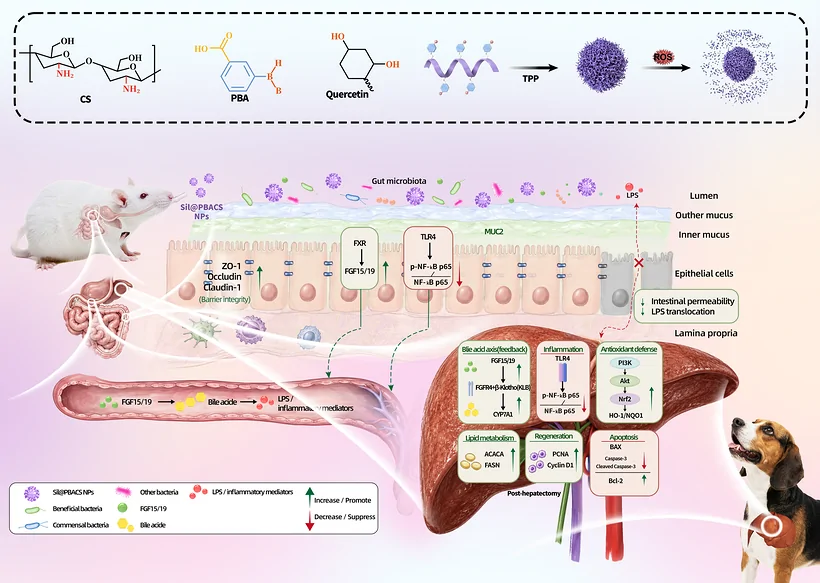

## 1 Introduction

Liver regeneration following hepatectomy is a complex physiological process that is critically influenced by the gut‐liver axis. It may be accompanied by severe oxidative stress, inflammation and metabolic disorders, and clinical treatment is hindered by the lack of effective multi‐target therapeutics [1]. The gut microbiome has become a key regulator of liver health through bidirectional communication via metabolic and immune responses [2]. However, clinical practices such as prophylactic antibiotic use during hepatectomy often lead to gut microbiota dysbiosis, which can impair the liver's capacity for regeneration and increase postoperative complications and mortality [3, 4]. Current literature suggests that gut‐derived microbes and their metabolites significantly contribute to slowing liver disease progression and promoting repair [5, 6, 7]. The bacterial phyla‐Firmicutes, Bacteroidetes, and Proteobacteria‐are essential for maintaining intestinal–hepatic homeostasis [8]. Recent studies have shown that microbial extracellular vesicles can alter intestinal microbial composition and exacerbate liver injury through systemic signaling [9]. These findings indicate that post‐hepatectomy recovery requires therapeutic strategies that can simultaneously attenuate hepatic injury and regulate gut‐liver axis disturbance. However, few oral delivery systems have been designed to respond to the oxidative and mildly acidic postoperative microenvironment while coordinating hepatic protection with gut microbiota‐ and metabolite‐associated recovery.

Silymarin (Sil), a flavonolignan complex extracted from milk thistle (*Silybum marianum*), has long been recognized for its hepatoprotective effects, attributed to its potent antioxidant, anti‐inflammatory, and antifibrotic activities [10, 11]. Clinically, silymarin has been used to treat a wide range of liver disorders, where it helps reduce hepatic enzyme levels, lipid accumulation, and oxidative stress [12, 13, 14]. Mechanistically, it enhances endogenous antioxidant defenses, such as superoxide dismutase (SOD) and total antioxidant capacity (TAOC), while suppressing reactive oxygen species (ROS) production, lipid peroxidation (malondialdehyde formation), and elevation of nitric oxide (NO) and lactate dehydrogenase (LDH) [15, 16, 17]. Sil has demonstrated therapeutic potential in inflammatory bowel disease (IBD) through inhibition of NF‐κB signaling and cytokine release [18], and regulation of specific microbial metabolites. Sil modulates 7‐ketodeoxycholic acid levels in bile acid metabolism to treat fatty liver disease and regulates glycated serum oxycholic acid derived from the intestinal microbiota to inhibit gallstone formation [19, 20]. However, its clinical translation remains limited by poor aqueous solubility, low oral bioavailability, and extensive first‐pass metabolism [21]. High doses may be required to achieve therapeutic efficacy, raising concerns of patient compliance and safety. Although lipid‐based nanoparticles and polymeric carriers have been explored to improve the pharmacokinetic profile of Sil, most systems do not address the post‐hepatectomy microenvironment, where oxidative stress, inflammation, and local pH changes may affect drug release and therapeutic efficacy. This limits their ability to provide responsive oral delivery for postoperative liver recovery.

Chitosan (CS) has good mammalian biocompatibility and antibacterial activity against *Escherichia coli* and *Staphylococcus aureus*. The ease of chitosan modification makes it a suitable carrier for controlled release and responsive delivery of drugs, nucleic acids, and proteins [22]. Chitosan‐based nanoparticles (Nps) have gained significant attention as drug delivery vehicles due to their intrinsic biocompatibility, biodegradability, muco‐adhesion, and antimicrobial activity [23]. Their cationic nature enhances interaction with negatively charged biological surfaces, improving mucosal adhesion and drug loading [24, 25]. Beyond its structural advantages, chitosan exhibits useful biological activities such as anti‐inflammatory effects and modulation of gut microbiota [26, 27, 28]. For example, selenized chitosan‐coated bismuth metal‐organic framework nanoparticles (Bi‐MOF@CS‐Se) have demonstrated gastric adhesion, potent antibacterial activity against *Helicobacter pylori*, and preservation of gut microbial balance [29]. Similarly, an oral bilirubin‐chitosan nanomedicine (LMWC‐BRNps) effectively attenuated hepatic inflammation and oxidative stress in a murine model of metabolic dysfunction‐associated steatohepatitis (MASH) [30]. Chitosan has also been shown to restore microbiota homeostasis by increasing the abundance of *Lactobacillus*, *Bacteroides*, and *Bifidobacterium* following antibiotic‐induced dysbiosis [31]. It is worth noting that phenylboronic acid‐grafted chitosan (PBACS) has emerged as a dual‐responsive nanocarrier capable of releasing drugs in response to low pH and to oxidative stress. By forming reversible boronate ester linkages with cis‐diol‐containing molecules like silymarin, PBACS offers stable encapsulation under physiological conditions and rapid release in the microenvironment of diseased tissue [32, 33, 34].

**SCHEME 1:**
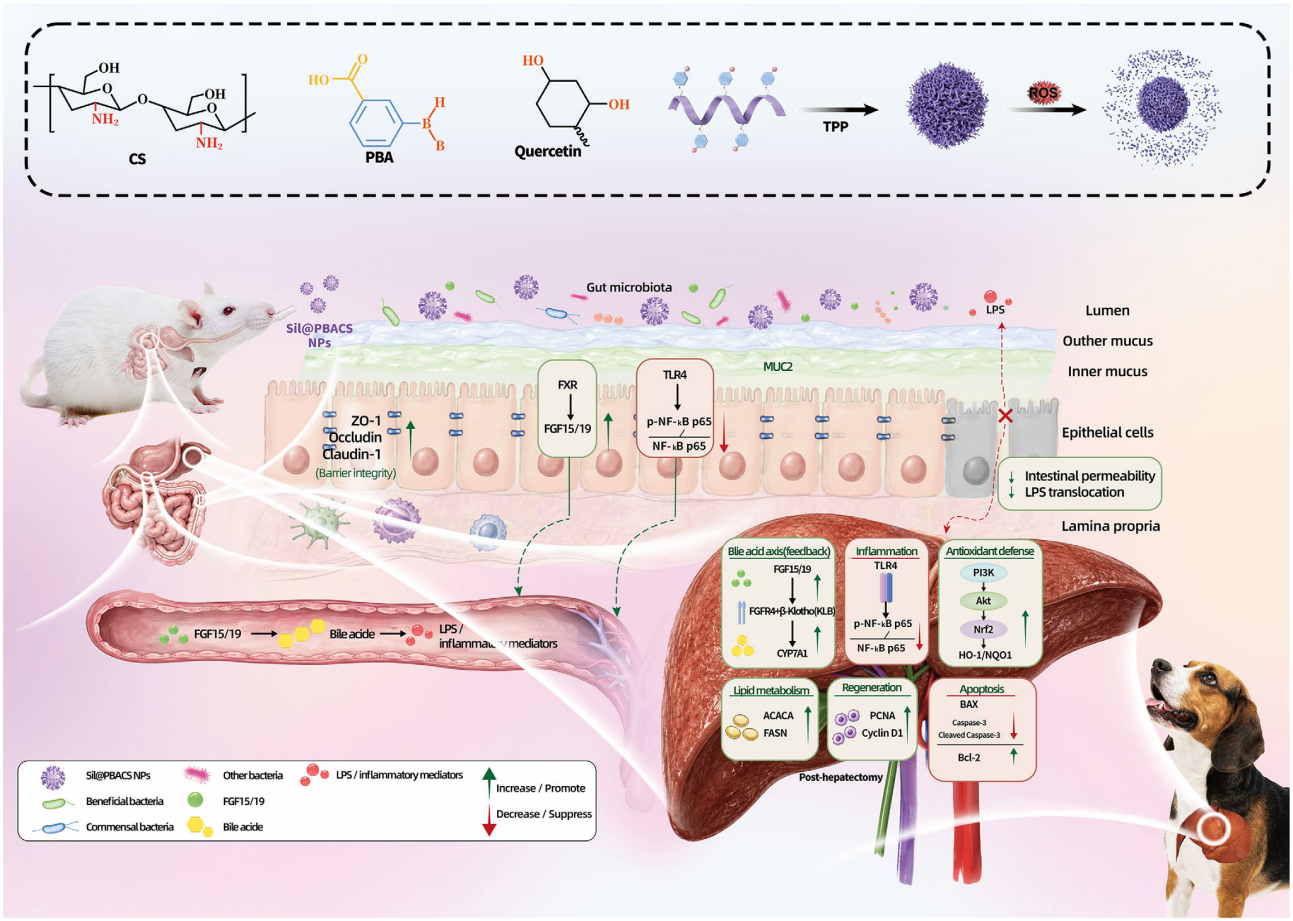
Schematic diagram of Sil@PBACS Nps‐mediated silymarin delivery for post‐hepatectomy hepatoprotection. The ROS/pH‐responsive phenylboronic acid‐chitosan nanoparticles enhanced silymarin encapsulation and lesion‐responsive release, thereby improving antioxidant protection and hepatic recovery after hepatectomy. Oral Sil@PBACS Nps remodeled gut microbiota and fecal metabolites, supported intestinal barrier‐ and bile acid‐related signaling, and regulated hepatic inflammatory, oxidative‐stress, lipid‐metabolic, apoptosis‐associated, and recovery‐related pathways through a gut–liver axis‐associated mechanism.

The canine hepatectomy model was used as a translational large‐animal model because it allows evaluation of Sil@PBACS Nps under clinically relevant surgical injury, perioperative stress, and postoperative liver recovery conditions. Taken together, the major knowledge gap is the lack of an oral nanoplatform that can improve Sil delivery, respond to the redox‐ and pH‐altered postoperative microenvironment, and link hepatic protection with gut‐liver axis‐associated regulation after hepatectomy. We hypothesized that silymarin‐loaded PBACS nanoparticles (Sil@PBACS Nps) could improve hepatic recovery following hepatectomy by enabling microenvironment‐sensitive drug release and modulating the gut‐liver axis. To test this hypothesis, we synthesized Sil@PBACS Nps via ionic gelation and characterized their physicochemical and biological properties. The nanoparticles exhibited high encapsulation efficiency, favorable biocompatibility, and robust antioxidant and antibacterial activity under acidic and oxidative conditions. To validate their in vivo efficacy, we employed a canine hepatectomy model and evaluated the effects of Sil@PBACS Nps on gut microbiota composition and fecal metabolite profiles. Quantitative liver proteomics further revealed significant changes in key metabolic and inflammatory pathways during postoperative hepatic recovery. Integrated microbiota, fecal metabolomics, hepatic proteomics, and targeted protein validation were used to explore the association between Sil@PBACS Nps treatment, gut–liver axis‐related responses, and hepatic recovery (Scheme 1). This study established a nanoplatform that couples pH/ROS‐responsive release with gut microbiota modulation. These findings support the potential of Sil@PBACS Nps as a responsive oral nanoplatform for post‐hepatectomy liver protection and provide a mechanistic framework linking material responsiveness, redox regulation, and gut–liver axis‐associated hepatic recovery.

## 2 Results and Discussion

### 2.1 Characterization

**FIGURE 1:**
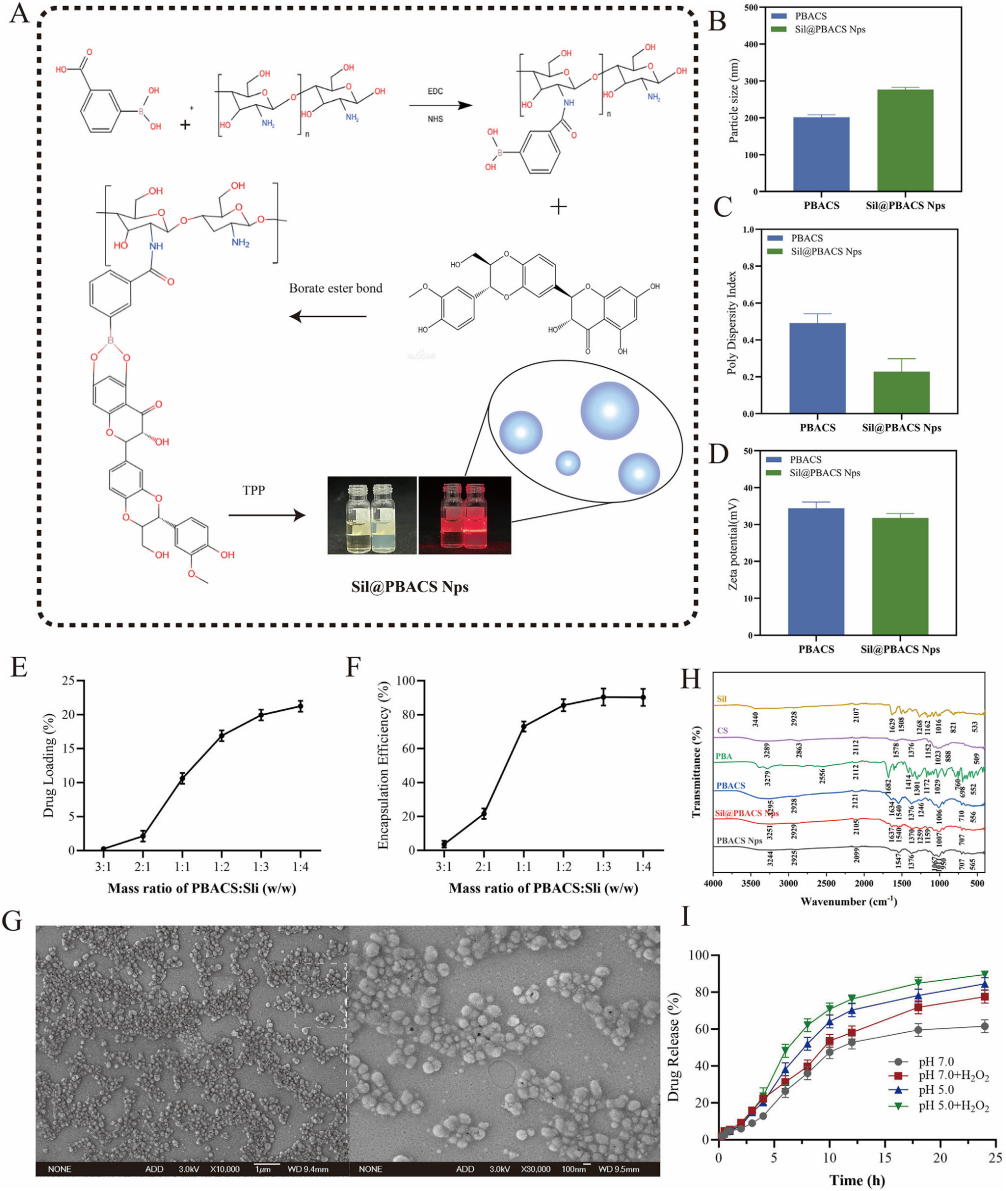
(A) Schematic diagram of the preparation method of Sil@PBACS. (B) Average particle size of Sil@PBACS Nps. (C) Polydispersity index of Sil@PBACS Nps. (D) Zeta potential of Sil@PBACS Nps. (E) The relationship between the Sli usage ratio and the encapsulation rate (F) and the drug loading rate. (G) SEM image of Sil@PBACS Nps at different magnifications Scale bars: 1 µm and 100 nm. (H) FTIR spectra of Sil@PBACS Nps and pure substance. (I) In Vitro Release Profiles of Sil@PBACS Nps.

The schematic diagram depicts the methodology employed for the preparation of Sil@PBACS Nps (Figure 1A). The optimal method and grafting rate of PBACS were as follows (see Supplementary Materials Figures S1–S4, Tables S1–S7). The formulation and processing parameters for nanoparticle preparation were optimized using single‐factor and response surface experiments based on the method reported by Yang et al. [35], with the optimization results presented in Figure S5, Tables S2–S7, and Figure S6 [36]. The optimal preparation conditions for nanoparticles were determined: the concentration of TPP was 1.25 mg/mL, the concentration of PBACS was 1 mg/mL, the stirring time was 45 min, and the ratio of drug to chitosan was 1:2. Under these conditions, through visual inspection and Tyndall effect analysis, it was preliminarily confirmed that the Sil@PBACS Nps were successfully formed. The particle size was 276.3 ± 5.4 nm (Figure 1B), and the PDI was 0.228 ± 0.07 (Figure 1C). The nanoparticles were evenly distributed and had good dispersibility, further confirming the stability and uniformity of the prepared nanoparticles. The zeta potential remained basically at 31.8 ± 1.2 mV (Figure 1D). The encapsulation efficiency of the prepared nanoparticles was 86.1 ± 0.2% (Figure 1E), the drug loading was 17.4 ± 0.1% (Figure 1F). The morphology of Sil@PBACS Nps was observed by scanning electron microscopy (SEM) (Figure 1G). It exhibited densely distributed, monodisperse spherical nanoparticles that were regular in shape, spherical with smooth surfaces and no obvious edges or protrusions. The surface of the nanoparticles was smooth, and no significant irregular crystal structure of free drugs was detected, indicating that silymarin was successfully encapsulated. In addition, the physical stability of the optimized Sil@PBACS Nps was monitored during long‐term storage. The nanoparticles could maintain structural stability at room temperature for up to 6 months, with no significant changes in particle size, polydispersity index (PDI), and zeta potential, indicating their long‐term dispersion stability (Table S8). However, after 6 months, it was observed that the particle size and PDI increased significantly, indicating that the nanoparticles aggregated and some structural degradation occurred. These results suggest that Sil@PBACS Nps can maintain physical stability for approximately 6 months under the test conditions, after which the colloidal stability begins to decline.

To examine the structural interactions among silymarin, phenylboronic acid‐grafted chitosan, and the formed nanoparticles, FTIR spectroscopy was performed (Figure 1H). The FTIR spectrum of free silymarin showed characteristic absorption bands at 1638 cm^−^^1^, corresponding to C = O stretching; 1512–1467 cm^−^^1^, assigned to aromatic C = C stretching; and 1267 cm^−^^1^, associated with C–O–C asymmetric stretching vibrations. In contrast, the FTIR spectrum of Sil@PBACS Nps revealed noticeable shifts in the positions and intensities of these bands, accompanied by the appearance of new absorption peaks, suggesting molecular interactions and structural rearrangement during nanoparticle formation. This finding differs from previous studies that reported non‐covalent drug encapsulation [37]. In the PBACS spectrum, several characteristic peaks of silymarin were retained, while new bands attributed to chemical modification were also observed. Notably, an absorption peak at 1366 cm^−^^1^ was assigned to the in‐plane bending vibration of the benzene ring, and a new signal at 1531 cm^−^^1^ was indicative of amide bond formation between the carboxyl group of phenylboronic acid and the primary amine of chitosan. These spectral changes, including peak shifts and new absorption bands, support PBACS modification and silymarin incorporation into the nanoparticle matrix, indicating the formation of a structurally stable Sil@PBACS Nps system.

### 2.2 In Vitro Release

Drug release was evaluated under neutral, acidic, ROS‐enriched, and combined acidic/ROS conditions to assess the responsiveness of Sil@PBACS Nps to key pathological features of the post‐hepatectomy microenvironment.

The in vitro release profile of Sil@PBACS Nps revealed a sustained‐release effect. As shown in Figure 1I, the release followed a biphasic pattern: an initial burst release phase followed by a prolonged sustained release over 24 h. The initial burst release can be attributed to silymarin molecules either adsorbed on the nanoparticle surface or loosely incorporated near the surface, allowing rapid diffusion into the release medium. This behavior aligns with the findings of [38], who reported a similar rapid release phase in nanoparticle systems. The subsequent sustained release phase is primarily driven by the gradual degradation or erosion of the chitosan nanoparticle matrix and the diffusion of encapsulated silymarin through the matrix. This release mechanism is consistent with previous studies on chitosan‐based nanoparticles [39, 40], where matrix degradation and diffusion were identified as key factors contributing to the extended drug release. At pH 7.0, in phosphate buffered saline (PBS) the cumulative release rate of silymarin from Sil@PBACS Nps after 24 h was 52.91 ± 3.25%. Medium supplemented with 1% H_2_O_2_ was used as the source of ROS to cleave the borate ester bonds and release silymarin from Sil@PBACS Nps [41] and the release rate reached 77.57 ± 3.5% after 24 h. Under acidic conditions (pH 5.0), the drug release in 24 h reached 84.56 ± 3.25%, and the highest rate (89.56 ± 3.55%) was reached in acidic 1% H_2_O_2_ (pH 5.0).

The drug release mechanism was investigated by fitting the release data into various kinetic models, and the R^2^ regression coefficients of the different models are depicted in Table 1. Release kinetic fitting was performed using mean cumulative release values obtained from three independent replicates. An R^2^ value close to 1 indicates a best fit for that model. The release data most closely fits Higuchi kinetics with R^2^ values of 0.94, suggesting that Sil@PBACS Nps possessed some slow‐release effects. These results highlight the dual functionality of Sil@PBACS Nps, providing a sustained‐release profile and demonstrating hydrogen peroxide responsiveness, underscoring their potential for controlled and stimulus‐responsive drug delivery in vitro. Although the acidic and H_2_O_2_‐containing media provide a simplified in vitro simulation of the post‐hepatectomy injury microenvironment, they cannot fully reproduce the dynamic in vivo conditions. Therefore, the release results should be interpreted as evidence of pH/ROS‐responsive behavior, while the actual in vivo release profile requires further pharmacokinetic and tissue‐distribution studies.

**TABLE 1:** Equation‐fitting results of Sil release from Sil@PBACS Nps.

Model	Fitting equation	R^2^adj
Zero‐order	Q = 4.62 + 2.98t	0.88
First‐order	Q = −231722.34(1−e1.42t)	0.86
Higuchi	Q = 14.14t1/2−11.26	0.94
Ritger‐Peppas	Q = 7.33t0.71	0.93

### 2.3 Antibacterial Activity

**FIGURE 2:**
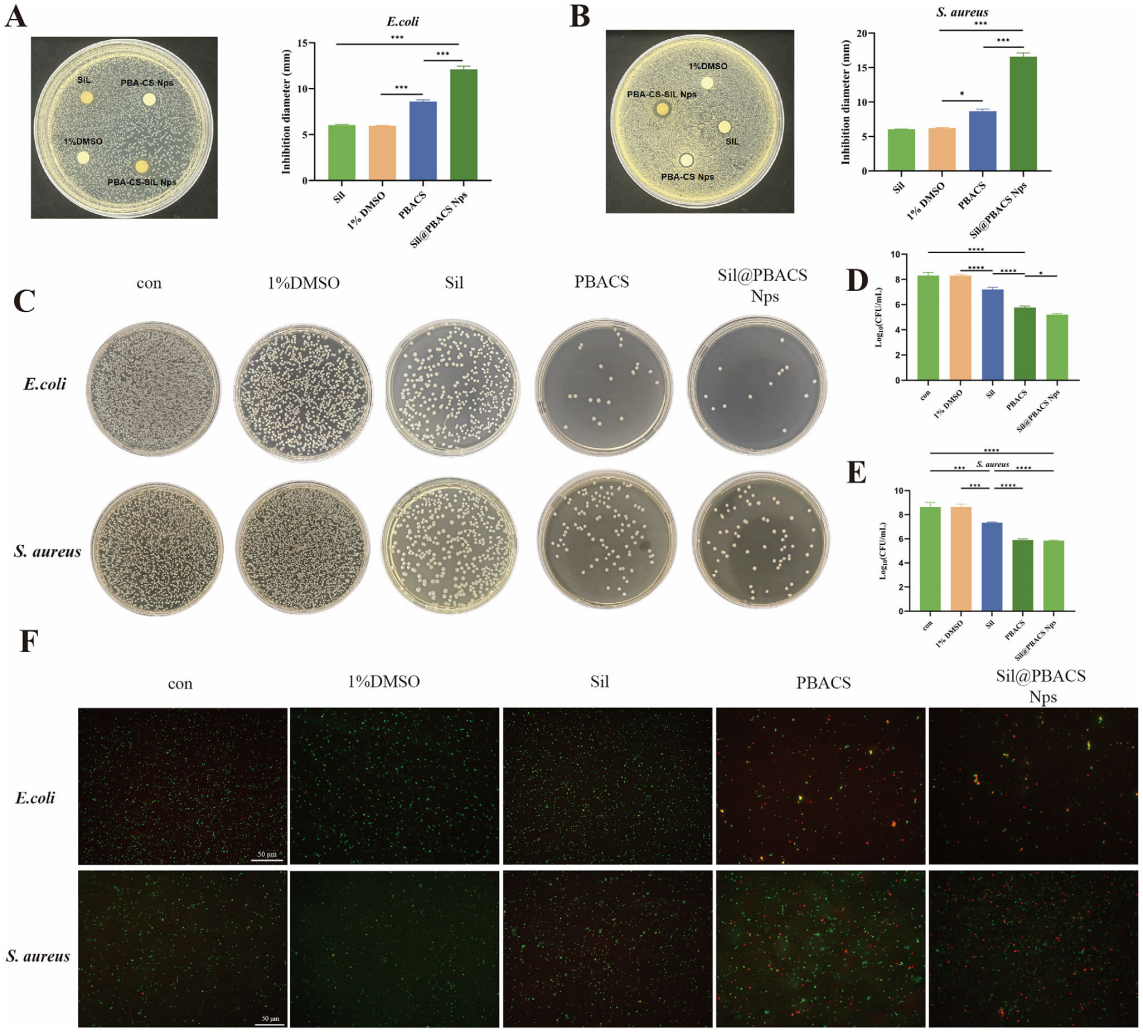
Inhibition zones and relative quantification of Sil@PBACS Nps against (A) *S. aureus* and (B) *E. coli*. (C) Bacterial colony formation of *S. aureus* and *E. coli* treated with different formulations. Corresponding bacterial number of (D) *S. aureus* and (E) *E. coli* determined by plate counting. (F) Live/dead vitality images of *E. coli* and *S. aureus* (Data are presented as mean ± SD; n = 3 independent samples in per group, *p < 0.05, **p < 0.01, ***p < 0.001, ***p < 0.001).

The antimicrobial activity of Sil@PBACS Nps was assessed by determining the minimum inhibitory concentration (MIC) and minimum bactericidal concentration (MBC) against *S. aureus* and *E. coli*, with results summarized in Table 2. Free silymarin showed no inhibitory activity against the Gram‐negative bacterium *E. coli*, consistent with its poor aqueous solubility and limited cell permeability. In contrast, Sil@PBACS Nps exhibited significant antibacterial effects, with MIC values of 0.125 mg/mL for *S. aureus* and 0.250 mg/mL for *E. coli*. The corresponding MBCs were 0.25 mg/mL and 0.50 mg/mL, respectively. Notably, the solvent control (1% DMSO) showed no inhibitory effect, confirming that the observed activity derived from the nanoparticle formulation. The improved antimicrobial efficacy of Sil@PBACS Nps is attributable to several combined carrier and drug effects. First, chitosan is known for its inherent antibacterial properties, which are significantly enhanced at the nanoscale due to increased surface area, stronger electrostatic interactions, and improved membrane adhesion [42]. Second, phenylboronic acid grafting enhances the interaction of chitosan with bacterial cell surfaces, further promoting membrane disruption and drug penetration. Third, encapsulation of silymarin within the PBACS matrix improves its solubility, protects it from degradation, and facilitates its sustained release at the infection site, all of which enhance its local antibacterial effect. These findings were further validated by inhibition zone assays (Figure 2A,B), which demonstrated larger inhibition zones for *S. aureus* (17.61 ± 0.31 mm) than for *E. coli* (12.71 ± 0.62 mm), aligning with MIC results and highlighting the preferential activity against Gram‐positive strains. This differential sensitivity may result from differences in cell wall structure, with Gram‐negative bacteria possessing an outer membrane that acts as a permeability barrier. The quantitative coating of the flat plate also showed similar results (Figure 2C–E). In addition, we conducted live/dead staining on the bacteria. The results of the dual fluorescence detection were consistent with the plate colony counting method, showing a similar trend. These results affirm the potential of Sil@PBACS Nps as a multifunctional antimicrobial delivery system for addressing bacterial infections, particularly where traditional phytochemicals show limited efficacy (Figure 2F).

**TABLE 2:** MIC and MBC of Sil@PBACS Nps Against *S. aureus* and *E. coli* (mg/mL, n = 3).

Group	*S. aureus*		*E. coli*	
	MIC (mg/mL)	MBC (mg/mL)	MIC (mg/mL)	MBC (mg/mL)
Sil	0.25	0.5	—	—
Sil@PBACS Nps	0.125	0.25	0.25	0.5
1% DMSO	—	—	—	—
PBACS	0.25	0.5	0.25	0.5

Compared with other nanocarrier systems, Sil@PBACS Nps exhibited superior antibacterial performance, with MIC values of 125 and 250 µg/mL, lower than those reported for unmodified chitosan nanoparticles (>200 µg/mL) [43] and previously described silymarin‐loaded nanoparticles, which are often limited by poor aqueous dispersion and reduced bacterial contact efficiency [44]. Moreover, the combined use of a functionalized chitosan shell and optimized nanoscale features promotes enhanced bacterial targeting, controlled drug release, and local accumulation, collectively contributing to improve bactericidal efficacy. In conclusion, Sil@PBACS Nps showed antibacterial activity against the representative Gram‐positive strain *S. aureus* and Gram‐negative strain *E. coli*, with stronger inhibitory effects than free silymarin under the tested conditions.

### 2.4 Biocompatibility

**FIGURE 3:**
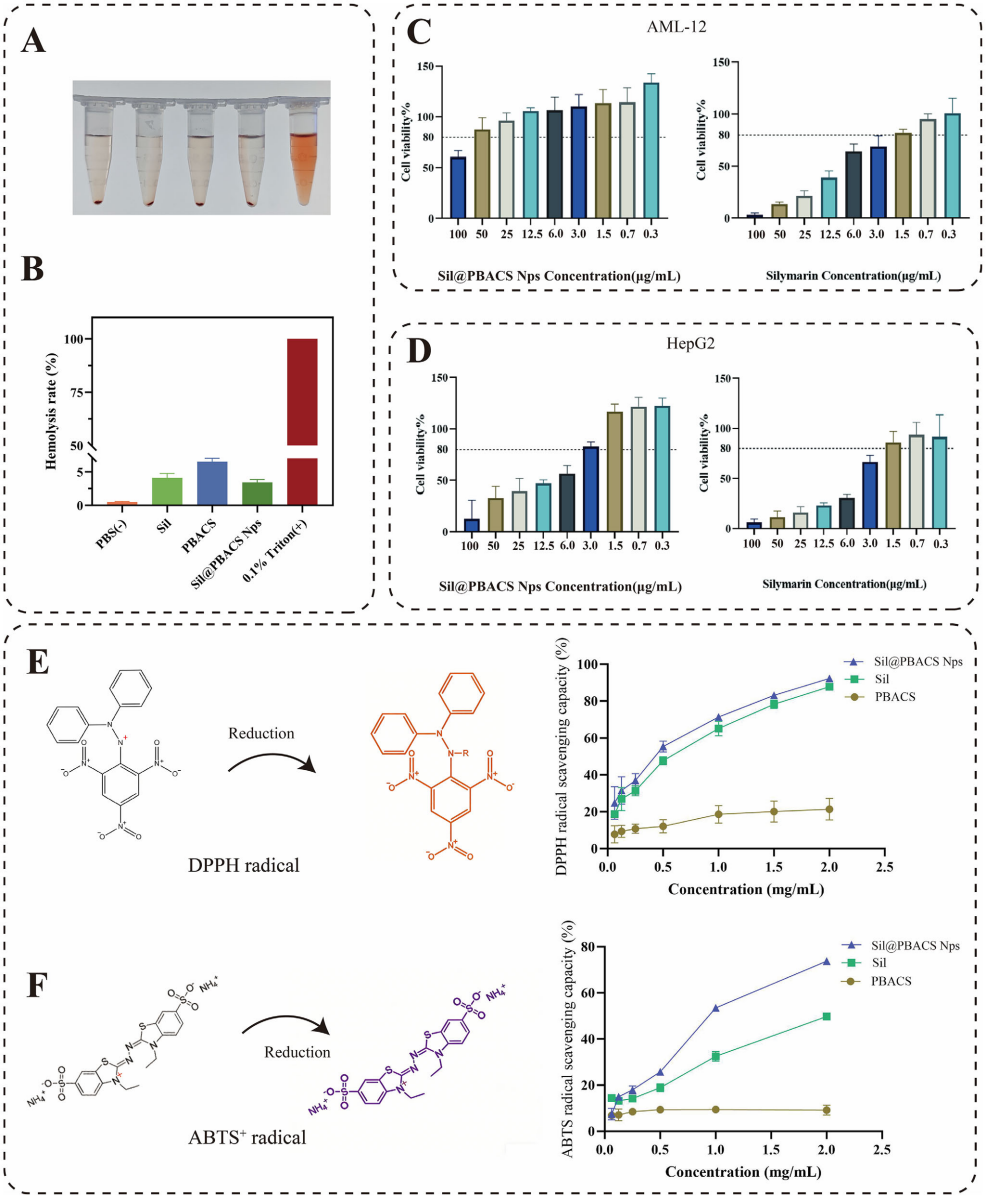
(A) Hemolysis experiment schematic diagram. (B) Hemolysis assay. “−” indicates the PBS negative control; “+” indicates the 0.1% Triton X‐100 positive control. (n = 3). Cytocompatibility of Sil@PBACS Nps: Viability of (C) AML‐12 cells and (D) HepG2 cells after 24 h incubation with various concentrations of Sil@PBACS Nps, assessed by CCK‐8 assay. (n = 3). In Vitro Antioxidant Activity of Silymarin Nanoparticles, (E) the principle of DPPH radical detection and the clearance rate of Sil@PBACS Nps and free silymarin. (n = 3). (F) The principle of ABTS radical detection and the clearance rate of Sil@PBACS Nps and free silymarin.

To evaluate the biomedical applicability of the developed nanocarriers, the biocompatibility of Sil@PBACS Nps was systematically assessed through hemolysis and cytotoxicity assays. The hemolysis test was performed using rabbit blood. Regardless of species difference, hemolysis is primarily governed by membrane interactions and osmotic principles that are conserved across mammalian species and rabbit blood has good sensitivity [45]. The rabbit model therefore provides a conservative and relevant safety assessment for human translation. All tested concentrations of Sil@PBACS Nps and the supernatant of PBS were almost colorless (Figure 3A) and exhibited hemolysis ratios below the internationally accepted 5% threshold (Figure 3B), indicating excellent blood compatibility. Notably, PBACS showed slightly higher hemolysis compared to Sil@PBACS Nps across all concentrations. This difference is likely due to the surface interaction between the cationic chitosan backbone and erythrocyte membranes. Upon silymarin encapsulation, the surface charge of the nanoparticles was partially neutralized [46], reducing electrostatic disruption and thereby improving hemocompatibility‐a phenomenon reported in similar systems where hydrophobic drug loading modulates surface activity and minimizes membrane damage.

Cytocompatibility was assessed using two cell lines: AML‐12 (murine hepatocytes) (Figure 3C) and HepG2 (human hepatocellular carcinoma cells) (Figure 3D). These were selected to represent both normal liver cells and hepatic tumor cells, as the liver is the primary target organ for silymarin‐related therapies. The use of both non‐tumorigenic and cancerous hepatic cells allowed for a broader understanding of cellular responses to the nanoparticle exposure. Cells were treated with Sil@PBACS Nps at concentrations ranging from 0.3 to 100 µg/ml for 24 h, and viability was quantified using the CCK‐8 assay. Sil@PBACS Nps maintained over 80% viability in AML‐12 cells up to 50 µg/mL, and over 85% in HepG2 cells at the same dose, suggesting low cytotoxicity and favorable compatibility across cell types.

### 2.5 In Vitro Antioxidant Activity

The in vitro antioxidant activity of Sil@PBACS Nps was evaluated using both DPPH and ABTS radical scavenging assays. At the maximum tested concentration (2.0 mg/mL), the DPPH scavenging rate reached 92.31 ± 0.22% (Figure 3E), while the ABTS scavenging rate was 73.78 ± 0.31% (Figure 3F). The scavenging rates for both radicals increased in a concentration‐dependent manner, indicating dose‐responsive antioxidant behavior.

In the case of Sil@PBACS Nps, silymarin is encapsulated within the phenylboronic acid‐grafted chitosan matrix, conferring both improved solubility and controlled release. During the DPPH assay, the slower kinetics allow time for the gradual diffusion of silymarin from the nanoparticle matrix, maintaining high scavenging efficiency over the assay duration. Conversely, the faster reaction kinetics in the ABTS assay primarily capture the contributions of silymarin molecules that are already exposed or rapidly released from the nanoparticle surface. This explains the more pronounced enhancement observed in the ABTS scavenging rate compared to free silymarin. Specifically, at 2.0 mg/mL, the DPPH scavenging rate of Sil@PBACS Nps (92.31 ± 0.22%) was only slightly higher than that of free silymarin (87.79 ± 0.61%), reflecting a cumulative antioxidant effect. However, the ABTS scavenging rate of the nanoparticle formulation (73.78 ± 0.31%) was significantly greater than that of free silymarin (49.74 ± 1.12%), indicating enhanced initial antioxidant property and reactivity toward fast‐reacting radicals.

This improvement in antioxidant performance can be attributed to multiple combined carrier and drug effects. First, chitosan itself exhibits intrinsic antioxidant activity, which may act in concert with the encapsulated silymarin to amplify overall scavenging efficiency [47]. Second, rather than increased surface area alone, the improved efficacy is more plausibly attributable to better aqueous dispersion, stabilization of bioactive molecules, and the sustained release profile imparted by the PBACS carrier. These characteristics increase the effective drug concentration in aqueous environments and prevent premature degradation or aggregation. Third, the nanocarrier system modulates the release kinetics of silymarin, allowing it to respond effectively under different radical conditions and time scales. This finding is consistent with previous research results [48], indicating that formulations based on nanocarriers can enhance the antioxidant activity of phytochemicals by increasing solubility, protecting the drugs from degradation, and promoting their interaction with ROS. Guo et al. [49], reported that the exosomes derived from wolfberry alleviated alcohol‐induced acute liver disease by altering the intestinal microbiota and its metabolite profile. This innovative perspective suggests a new approach for treating liver damage through the gut‐liver axis.

### 2.6 Preliminary Comparative Evaluation of Murine Model

**FIGURE 4:**
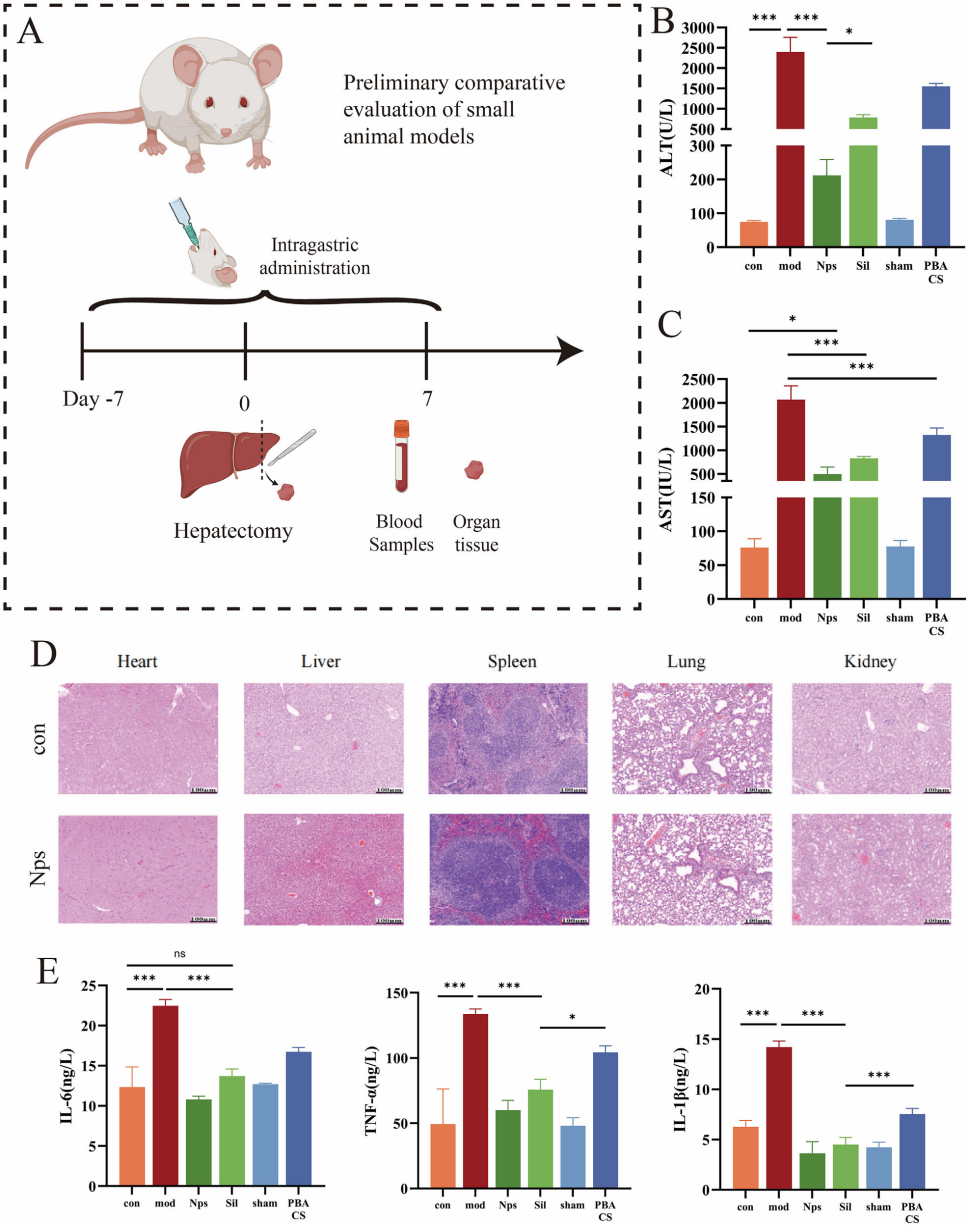
(A) Schematic of the experimental design. (B,C) Serum ALT and AST levels on day 7 post‐hepatectomy. (D) Representative H&E staining of major organs for biosafety evaluation on day 7. (E) Serum inflammatory cytokines on day 7. (con): without any treatment; (mod): hepatectomy model group; (Nps): hepatectomy + Sil@PBACS Nps group (Sil): hepatectomy + free silymarin group. Sham operation group (Sham): with incision of abdominal wall but suture without drug treatment and (PBACS): hepatectomy + PBACS group (Data are presented as mean ± SD; n = 6 biologically independent animals per group, *p < 0.05, **p < 0.01, ***p < 0.001).

Female SPF‐grade Kunming mice (6–7 weeks old, 20–25 g) were obtained from Sibefu (Beijing) Biotechnology Co., Ltd (license No. 110324241107287862) and acclimated for 1 week prior to experimentation. All procedures were approved by the Animal Ethics Committee of Beijing Agricultural University. In order to systematically compare the effects of each component of the preparation, mice were randomly divided into six groups: control group (con), sham operation group (sham), hepatectomy model group (mod), hepatectomy + PBACS group (PBACS), hepatectomy + free silymarin group (Sil) and hepatectomy + Sil @ PBACS Nps group (Nps) (Figure 4A). Treatments were administered by gavage for 7 days prior to surgery; Sham groups received saline. A partial hepatectomy (left lateral lobe resection) was performed following procedures analogous to those used in dogs. Blood samples were collected on day 7 post‐surgery Compared with the mod group, the group treated with Sil@PBACS Nps had markedly reduced serum ALT (Figure 4B). And AST (Figure 4C) levels and less hepatic injury by histopathological examination. ALT levels were decreased relative to the free Sil group. Histological analysis demonstrated that treatment with Sil‐loaded Nps alleviated hepatocellular swelling, sinusoidal dilation, and inflammatory infiltration; no evident pathological abnormalities were observed in major organs, supporting acceptable biosafety (Figure 4D). In addition, Sil@PBACS Nps significantly suppressed pro‐inflammatory cytokines (IL‐6, TNF‐α, IL‐1β) compared with both the PBACS and free silymarin groups, whereas the PBACS alone exhibited minimal activity (Figure 4E). The relative contributions of free silymarin, the carrier effect, and carrier‐drug synergy require confirmation in future large‐animal studies with an expanded group design. These results indicate that the experimental design in the murine model incorporated both drug and carrier controls and demonstrated the superior therapeutic efficacy of the Sil@PBACS Nps formulation.

### 2.7 Biological Distribution of Sil@PBACS Nanoparticles and Restore Redox Homeostasis After Hepatectomy

**FIGURE 5:**
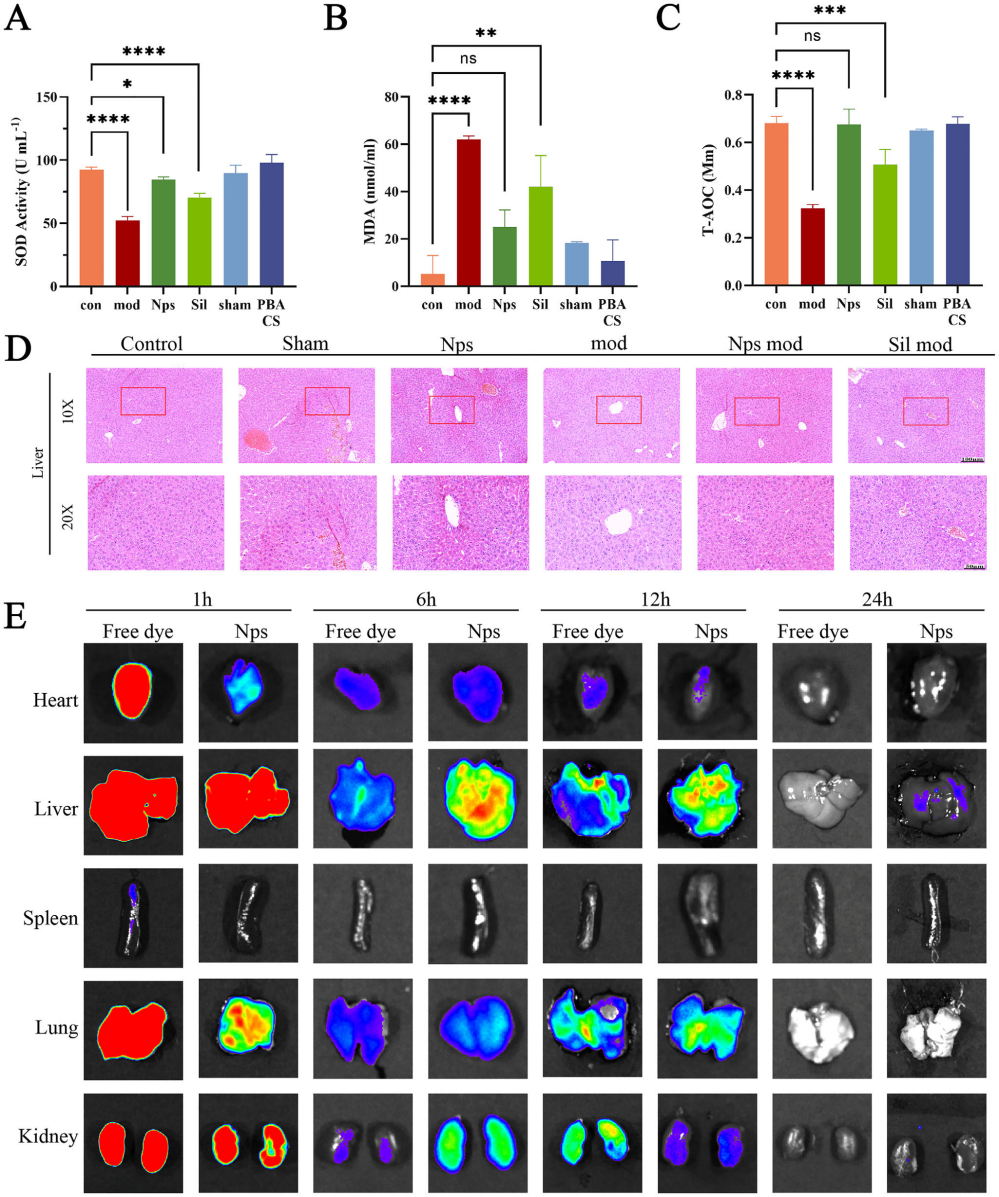
Antioxidant evaluation, liver histopathology, and ex vivo biodistribution of Sil@PBACS Nps (Nps) after hepatectomy. (A) SOD. (B) MDA. (C) T‐AOC. (D) Representative H&E staining of liver tissues. (E) Ex vivo fluorescence imaging of the heart, liver, spleen, lung, and kidney at 1, 6, 12, and 24 h after administration of Free dye or Nps, showing the biodistribution and hepatic retention of the nanoparticle formulation. (con): without any treatment; (mod): hepatectomy model group; (Nps): hepatectomy + Sil@PBACS Nps group (Sil): hepatectomy + free silymarin group. Sham operation group (Sham): with incision of abdominal wall but suture without drug treatment and (PBACS): hepatectomy + PBACS group (Data are presented as mean ± SD; n = 3 biologically independent animals per group, *p < 0.05, **p < 0.01, ***p < 0.001, ****p < 0.0001).

Efficient hepatic retention is critical for converting the intrinsic antioxidant activity of silymarin into sustained protection of the injured liver. Hepatectomy markedly reduced SOD activity and T‐AOC levels while increasing MDA accumulation (Figure 5A–C), reflecting weakened antioxidant defense and enhanced lipid peroxidation. Free silymarin partially attenuated these changes, whereas Sil@PBACS Nps produced a stronger antioxidant response, as indicated by greater recovery of SOD activity and total antioxidant capacity together with lower MDA levels. These results indicate that nanoparticle‐mediated delivery amplified the antioxidant efficacy of silymarin and reduced oxidative stress‐associated liver injury. Sil@PBACS Nps effectively restored hepatic redox balance. Histological examination further confirmed the tissue‐protective effect of Sil@PBACS Nps (Figure 5D). The Control, Sham, and Nps groups maintained well‐organized hepatic architecture, whereas the injury group exhibited hepatocyte swelling, sinusoidal congestion, inflammatory infiltration, and structural disruption. Free silymarin alleviated part of these lesions, but Sil@PBACS Nps achieved more evident preservation of hepatic morphology, with reduced inflammatory cell infiltration and milder parenchymal damage. Ex vivo fluorescence imaging showed that the Nps exhibited stronger and more sustained hepatic fluorescence signals compared with the Free dye (Figure 5E). Although both formulations were detectable in major organs after administration, the fluorescence signal of Free dye declined rapidly, whereas Nps produced more persistent hepatic signals from 1 to 24 h. This prolonged fluorescence retention suggests that PBACS‐based nanoparticle construction extended the residence time of the fluorescently labeled carrier within the liver, indicating the potential of this formulation to enhance local retention of encapsulated cargo. The time‐dependent fluorescence enhancement also indicates improved hepatic accessibility of the Nps, although direct evidence of sustained silymarin exposure requires further pharmacokinetic validation. Collectively, the fluorescence imaging data support that the PBACS‐based formulation confers a liver‐retentive property to the fluorescent carrier, which may contribute to the observed improvements in oxidative stress markers and tissue architecture. These findings, combined with the biochemical and histopathological evidence, support Sil@PBACS Nps as a promising nanoplatform for hepatoprotection after hepatectomy.

However, this assay reflects the distribution of the fluorescently labeled carrier and does not directly quantify silymarin exposure. Direct evaluation of silymarin delivery requires validated HPLC or LC–MS/MS analysis of silymarin or its active components in plasma, liver tissue, intestinal contents, and feces. Therefore, the present study supports improved formulation performance, stimuli‐responsive release, and hepatoprotective efficacy of Sil@PBACS Nps, but does not establish a complete pharmacokinetic profile of silymarin after nanoparticle administration.

### 2.8 Sil@PBACS Nps Reduced Post‐Hepatectomy Liver Injury

**FIGURE 6:**
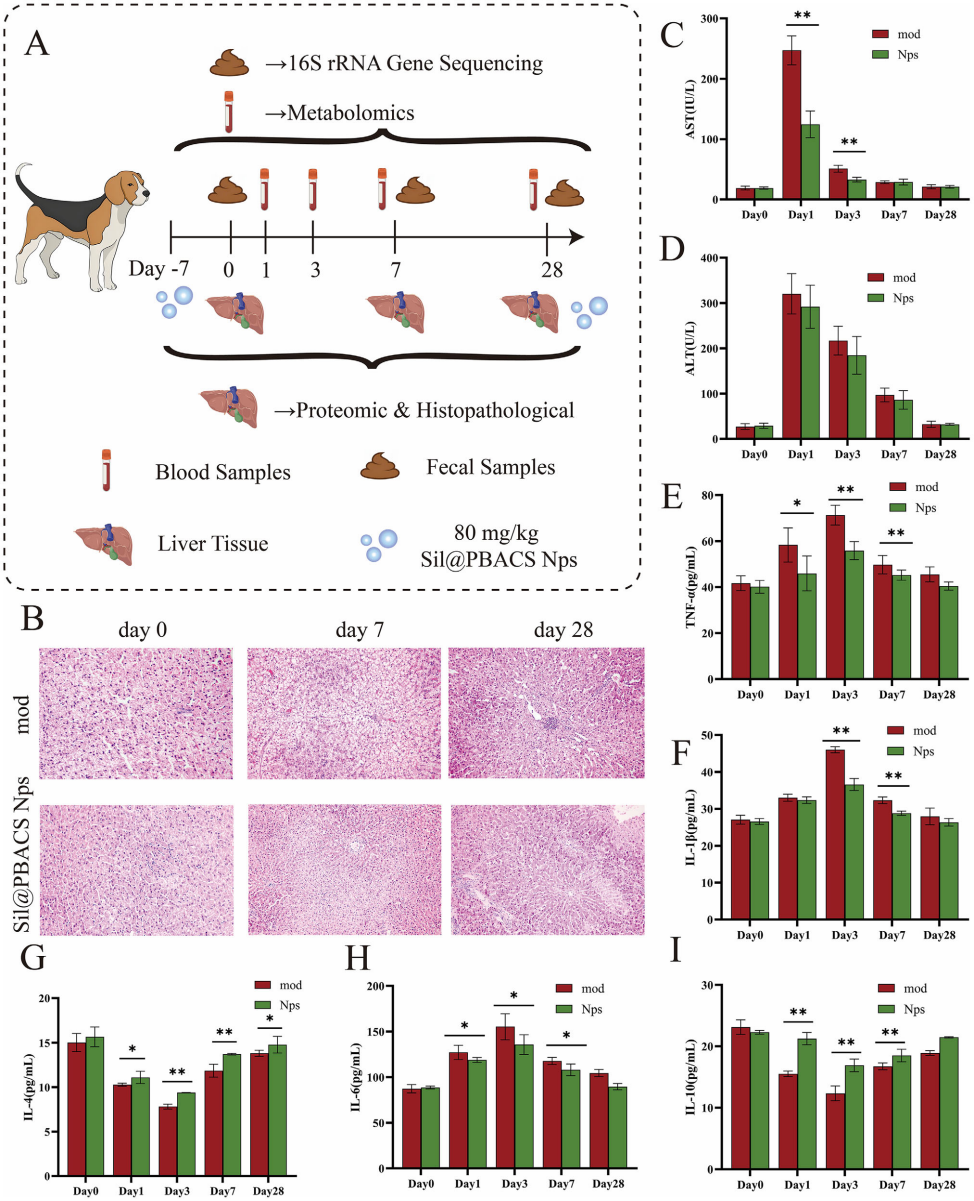
(A) Experimental design of liver injury in dogs after hepatectomy. (B) Histopathological examination of liver sections was performed after H&E staining (200×). (C–I) Effects of Sil@PBACS Nps on liver function and inflammatory cytokines.

The experimental workflow for the canine hepatectomy model is shown in Figure 6A, and representative H&E staining is presented in Figure 6B. In the model group at day 7 (mod7), lobular hepatocytes exhibited pronounced vacuolar degeneration, focal necrosis, disrupted hepatic cord organization, and marked inflammatory cell infiltration. In contrast, the Sil@PBACS Nps group at day 7 (Sil@PBACS Nps 7) showed substantially preserved hepatic architecture, with well‐defined hepatic cords, absence of vacuolar degeneration, and minimal inflammatory infiltration. By day 28, residual hepatocellular degeneration with sporadic inflammatory foci persisted in the model group (mod 28), whereas the treated group (Sil@PBACS Nps 28) displayed oval cell proliferation and bile duct hyperplasia in the portal region, accompanied by orderly hepatic cords and no detectable hepatocyte necrosis. These histological findings indicate enhanced hepatic repair following treatment.

To corroborate the histological observations, serum biochemical and inflammatory indices were assessed. Relative to baseline, ALT and AST activities increased significantly after hepatectomy at all‐time points (*p <*0.01), confirming successful model establishment (Figure 6C–I). Compared with the model group, AST activity was significantly lower in the treated group on days 1 and 3 (*p <*0.01). On day 1, TNF‐α and IL‐6 levels were reduced, whereas IL‐10 and IL‐4 levels were elevated in the treated group. This anti‐inflammatory shift persisted on days 3 and 7, with decreased TNF‐α, IL‐1β, and IL‐6 and increased IL‐4 and IL‐10 relative to the model group.

Previous studies have demonstrated that orally administered silymarin can enhance liver function and immunity, reduce blood lipid levels (triglycerides, TG), improve liver function (AST and ALT), and decrease lipid accumulation in the livers of mice in vivo [50]. Furthermore, silymarin ameliorating acetaminophen‐induced liver oxidative damage. Additionally, significantly lowered serum lipid levels, hepatic lipid accumulation and pro‐inflammatory cytokines, thereby ameliorating a mice model of nonalcoholic fatty liver disease [51]. Consistent with these findings, our study showed that Sil@PBACS Nps decreased ALT and AST levels and improve liver function, indicating that Sil@PBACS Nps protected the animals from liver injury. Levels of TNF‐α, IL‐6, and IL‐1β were significantly lower, while levels of IL‐4 and IL‐10 were significantly higher in the Sil@PBACS Nps group, indicating that Sil@PBACS Nps acts as a potent anti‐inflammatory agent following hepatectomy in the canine model While the Sil@PBACS platform demonstrated robust efficacy in the canine model, the current study design precludes an unequivocal separation of the drug's individual effects from the carrier's potential contribution or combined carrier and drug effects, prioritizing translational performance in a large‐animal surrogate. Future studies utilizing a multi‐arm design including empty carrier and free drug controls, are necessary to fully map the mechanistic landscape of this synergy.

Note: Data shown are mean ± standard deviation, n = 6. D0, pre‐operation; D1, post‐operative day 1; D3, post‐operative day 3; D7, post‐operative day 7. Comparison at different times before the operation (day 0), ^##^ indicates *p <* 0.01, a highly significant difference and ^#^ indicates *p* < 0.05, a significant difference. The mod and Sil@PBACS Nps groups of the two groups at the same time point were compared. ^**^ represents *p* < 0.01, a highly significant difference, and ^*^ represents 0.01< *p* <0.05, a significant difference. AST, aspartate transferase; ALT, alanine transaminase; TNF, tumor necrosis factor; IL, interleukin.

### 2.9 Sil@PBACS Nps Optimizes the Gut Microbiota Composition Following Hepatectomy

**FIGURE 7:**
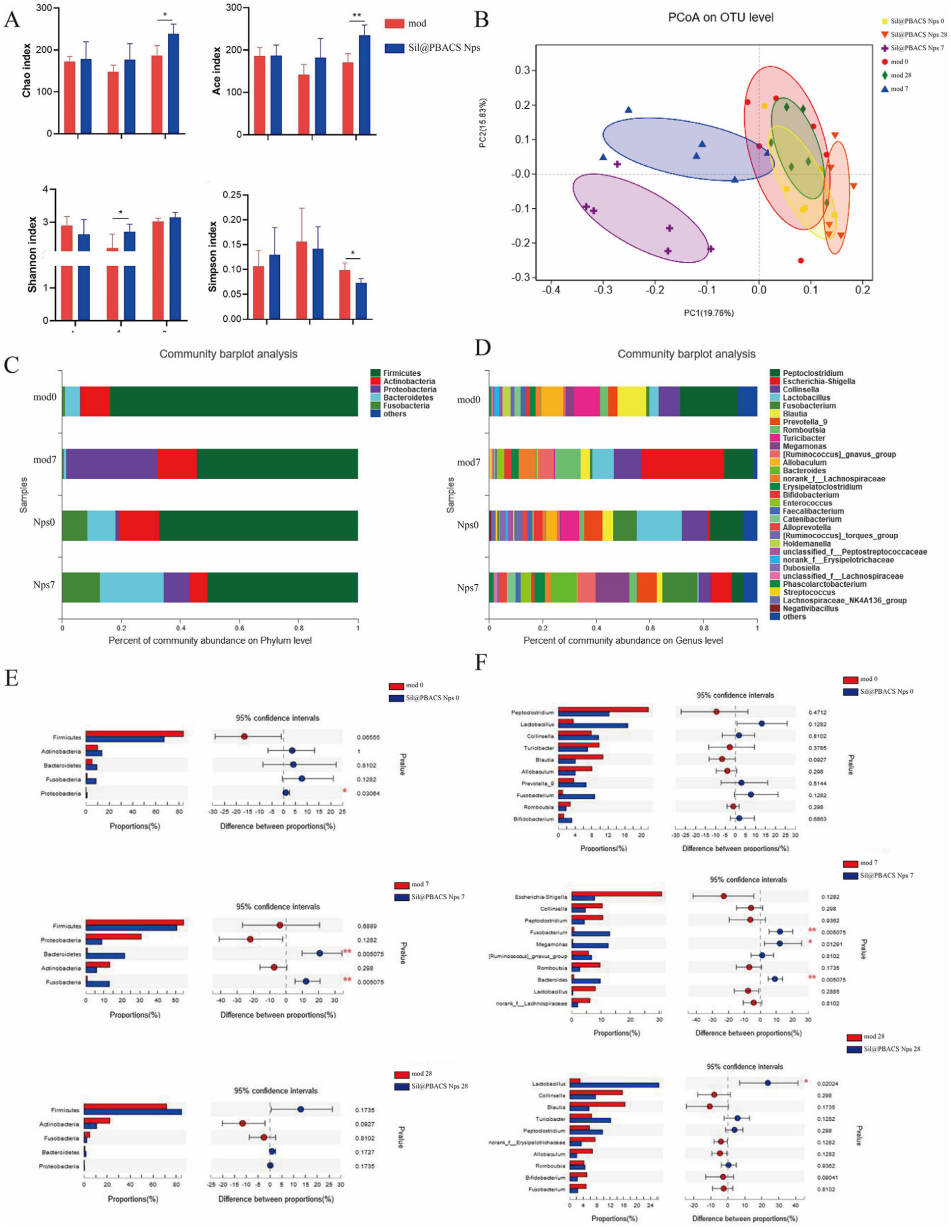
(A) The Chao, ACE, Shannon, and Simpson indices were derived from alpha diversity analysis among the two groups (n = 6). (B) PCoA of Bray‐Curtis distances derived from β‐diversity analysis. (C) Bacterial taxonomic profiling of fecal bacteria at the phylum (top 5) and (D) genus levels (*p < 0.05, **p < 0.01). (E) Differences between the relative abundances of five predominant bacterial phyla in fecal samples between two groups at various time points. (F) Differences between the relative abundance of the ten predominant bacterial genera in fecal samples between two groups at various time points. The extended error bar plot was generated using STAMP software. Welch's two‐sided test was used, and Welch's inverted test value was 0.95.

In the subsequent analysis, we examined the impact of Sil@PBACS Nps on fecal microbiota composition post‐hepatectomy. Notably, in comparison to the mod7 group, the Shannon index showed a significant increase (*p <*0.01), while the Simpson index exhibited a significant decrease (*p <*0.05) in the Sil@PBACS Nps 7 group. These findings suggest that Sil@PBACS Nps enhances the diversity of the intestinal bacterial community. Additionally, the ACE and Chao indices were markedly elevated in the Sil@PBACS Nps 28 group relative to mod28 (*p* < 0.01) (Figure 7A). Principal Coordinates Analysis (PCoA) based on Bray‐Curtis distances further revealed a distinct shift in microbial diversity at the OTU level following Sil@PBACS Nps supplementation (stress = 0.076, R = 0.319, *p* = 0.018; Figure 7B).

Remarkably, Sil@PBACS Nps 7 showed a significant increase in the relative abundance of Fusobacteria and Bacteroidetes compared to mod7 (*p <* 0.01) (Figure 7C,E). At the genus level, Sil@PBACS Nps7 demonstrated elevated levels of *Fusobacterium* (*p* < 0.01), *Bacteroides* (*p* < 0.01), and *Megamonas* (*p <* 0.05) in comparison to mod7 (Figure 7D,F). It is noteworthy that the phylum Bacteroidetes comprises a large group of dominant commensal bacteria, with the genus Bacteroides known for generating short‐chain fatty acids (SCFAs) [52]. *Bacteroides* genera produce butyric acid, which plays a protective role in maintaining the integrity of the intestinal barrier [53]. While previous studies have identified a connection between postoperative hepatic recovery post‐partial hepatectomy and intestinal microbiota [54, 55], the underlying mechanisms of communication between gut microbiota and extra‐intestinal organs remain largely unexplored. Interestingly, it has been reported that intestinal microbiota is closely associated with the process of 30% hepatectomy in mice, where a shift from Firmicutes to Bacteroidetes in stool bacterial composition was observed. In this study, Sil@PBACS Nps notably increased the relative abundance of Bacteroidetes. However, given the context‐dependent nature of these taxa, such increases should not be automatically interpreted as beneficial without further functional characterization. *Fusobacterium genera* can maintain the microecological balance within a certain abundance range but have also been significantly associated with colorectal cancer, necrotizing colitis and other diseases. Consistent with previous research, chitosan microparticles have been shown to be closely related to the relative abundance of *Fusobacterium* [56]. Moreover, prior studies have revealed that *Fusobacterium* and *Megamonas* are highly abundant in the gut microbiota of healthy dogs but are significantly changed in dogs affected by canine atopic dermatitis [57]. Additionally, it has been reported that chitosan microparticles maintain strong antimicrobial activity against pathogenic bacteria in cow uteri, demonstrating their potential as an alternative antimicrobial agent. Similarly, it has been demonstrated that increased abundance of certain taxa, including *Megamonas*, *Bacteroides*, and *Fusobacterium* within the gut microbiota may suppress pathogenic microorganisms and promote the production of short‐chain fatty acids in certain contexts [58]. In both in‐vitro and in‐vivo studies conducted by Wu [59], *Megamonas rupellensis* was shown to possess genes for myo‐inositol degradation, and the addition of myo‐inositol effectively inhibited fatty acid absorption in intestinal organoids, enhancing lipid absorption and obesity, thus suggesting potential strategies for future obesity management. Overall, the observed increase in the relative abundances of *Bacteroides*, *Fusobacterium* and *Megamonas* during the postoperative period may be related to the intestinal barrier function. This warrants further investigation, particularly regarding their potential impact on alleviating liver damage caused by bacterial translocation.

**FIGURE 8:**
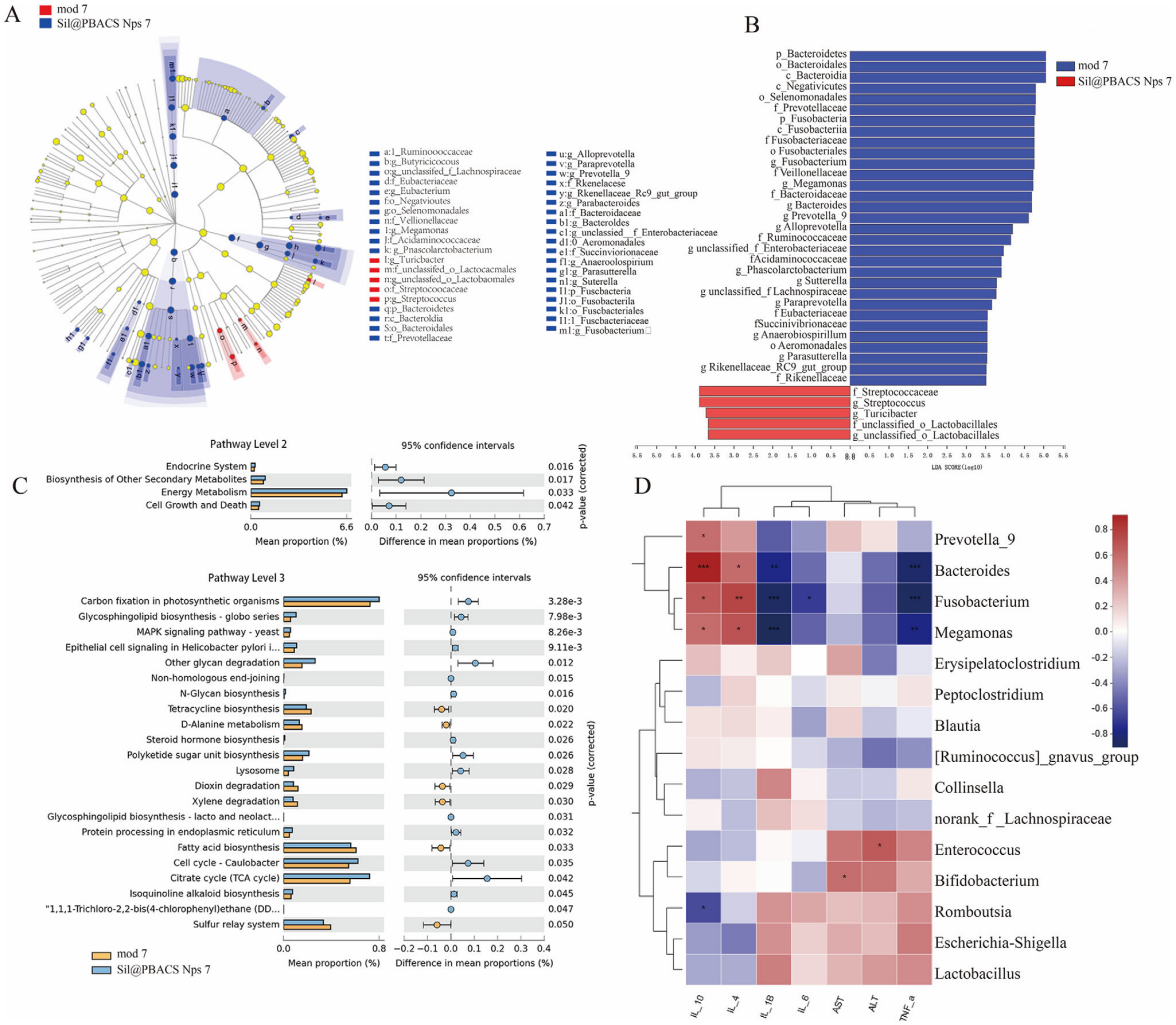
(A,B) LEfSE analysis of the effect of Sil@PBACS Nps on fecal bacteria (from phylum to genus) between Sil@PBACS Nps 7 and mod7. (C) Functional prediction of differential bacteria between Sil@PBACS Nps7 and mod7 groups based on PICRUSt2. (D) The Spearman correlations between the significantly differential microbiota at the genus level and inflammatory and liver function indices. The genera was selected from the significantly differential microbiota top 15. ^*^Represents the correlation *p* < 0.05, ^**^ *p* < 0.01, and ^***^ *p* < 0.001.

LEfSe analysis identified that the genera *Fusobacterium*, *Megamonas*, *Bacteroides*, and *Prevotella_9* were significantly enriched following Sil@PBACS Nps treatment, whereas the genera *Streptococcus*, *Turicibacter*, and *g_unclassified_o_Lactobacillales* were more abundant in the mod group (Figure 8A,B). Functional prediction using PICRUSt2 revealed that the Sil@PBACS Nps group showed significant enhancement in pathways related to the cell cycle, specifically in the cell cycle‐Caulobacter, the citrate cycle (TCA cycle), and carbon fixation in photosynthetic organisms, while fatty acid biosynthesis was notably reduced (Figure 8C). Moreover, Spearman correlation analysis demonstrated a significant relationship between the most abundant bacterial genera and inflammatory factors in serum within the Sil@PBACS Nps 7 and mod7 groups. *Bacteroides*, *Fusobacterium*, and *Megamonas* were positively correlated with anti‐inflammatory cytokines IL‐10 and IL‐4 and negatively correlated with pro‐inflammatory cytokines IL‐1β and TNF‐α (Figure 8D). Gao et al. [60] also pointed out that the inflammatory response and the intestinal microbiota play a crucial role in the occurrence and development of liver disease. Our findings suggest that the Sil@PBACS Nps‐induced reduction in post‐hepatectomy liver dysfunction is associated with changes in the intestinal microbiota; however, because our sequencing was based on the V3‐V4 region of 16S rRNA, taxonomic resolution was limited and the functional differences at the species or strain level could not be resolved. Furthermore, it should be noted that the PICRUSt2 and random forest analyses are still methods for generating hypotheses, rather than for inferring or validating the results. Some studies have also shown that isolates of *Fusobacterium* preferentially co‐aggregate with certain specific strains [61]. Due to this interaction, species in the *Fusobacterium* genus can be mutualists, facilitators, antagonists and/or synergists.

### 2.10 Sil@PBACS Nps Alters the Fecal Sphingolipid Metabolism After Hepatectomy

**FIGURE 9:**
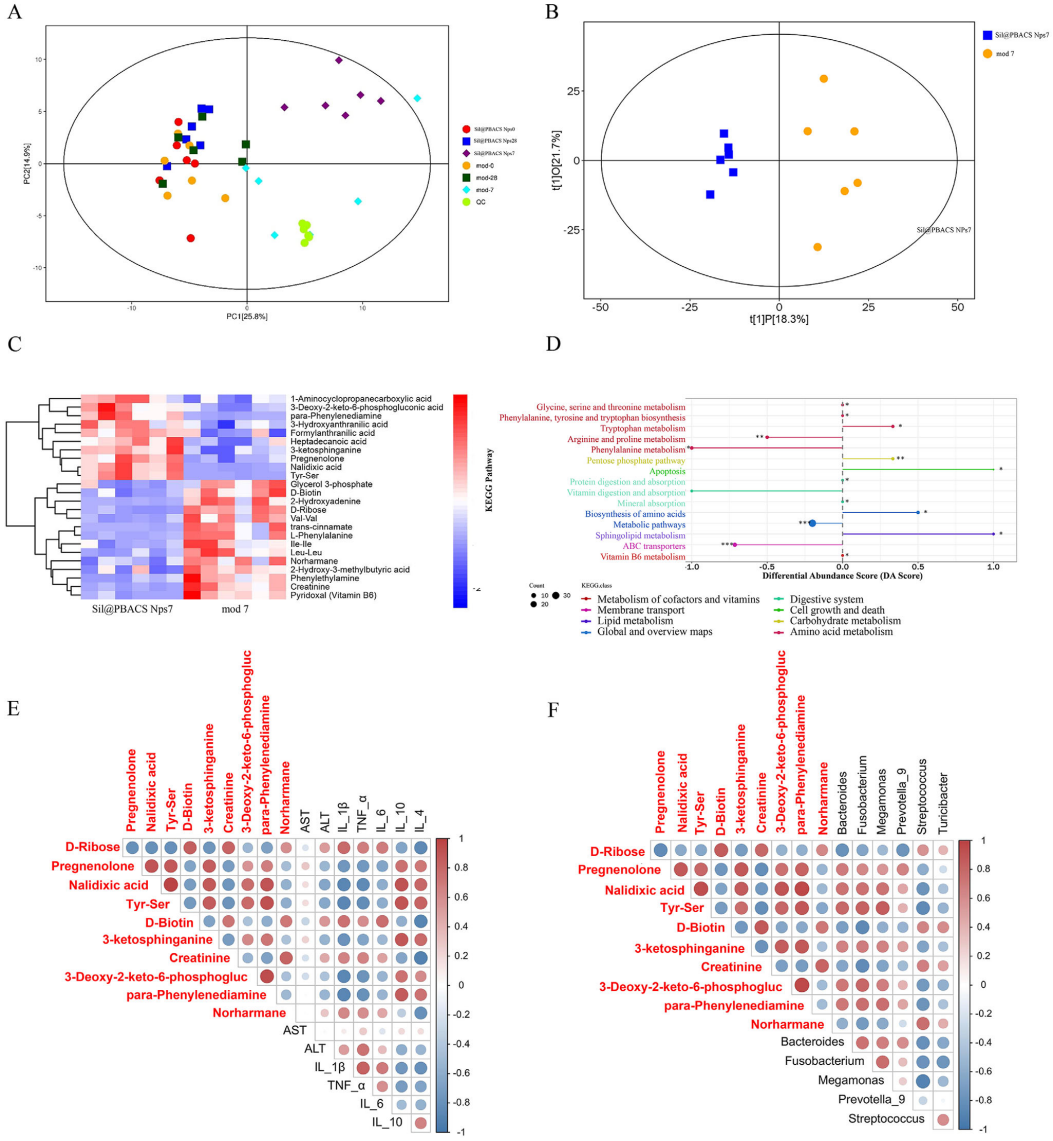
Fecal metabolomics analysis in response to chitosan supplementation in canines with postoperative liver injury. (A) Principal Component Analysis (PCA), (B) Orthogonal Partial Least Squares Discriminant Analysis (OPLS‐DA), and (C) cluster heatmap showing fecal metabolites differentially expressed between the Sil@PBACS Nps7 and mod7 groups (*p* < 0.01). (D) KEGG pathway enrichment analysis of the differentially expressed metabolites. Spearman's correlation analysis was performed to assess the relationships between the top ten altered fecal metabolites and liver function as well as inflammatory factors (E), and with the top six fecal microbiota species (F).

The effect of Sil@PBACS Nps on host fecal metabolites was assessed using liquid chromatography‐mass spectrometry (LC‐MS). Principal Component Analysis (PCA) of the fecal samples (Figure 9A), demonstrated distinct clustering. Further analysis using OPLS‐DA score plots (Figure 9B and Figure S8) revealed clear separation between the Sil@PBACS Nps7 and mod7 groups on day 7, indicating significant alterations in fecal metabolites. A total of 169 differentially expressed (DE) metabolites were identified between these groups, with a heatmap highlighting the 24 most promising potential biomarkers (*p <*0.01, Figure 9C, Table S9). These differential metabolites were predominantly enriched in sphingolipid metabolism and the pentose phosphate pathway (Figure 9D). Microbiota‐derived metabolites have been shown to influence host metabolism and intestinal health [62]. We found that the pentose phosphate pathway and sphingolipid metabolism were markedly altered following Sil@PBACS Nps treatment. The pentose phosphate pathway produces a large amount of the reducing species NADPH, which is necessary to suppress oxidative damage by maintaining glutathione in a reduced state in erythrocytes [63].

This aligns with our previous findings, where intramammary infusion of matrine‐chitosan hydrogels significantly impacted metabolic pathways in sub‐clinical mastitis, particularly those related to glycerophospholipid and sphingolipid metabolism, consistent with the known antimicrobial and anti‐inflammatory properties of matrine. Prior studies also demonstrated that chitosan and silymarin significantly affect gut bacterial metabolism by altering pathways involved in tryptophan, indole, bile acid, tyrosine, and phenol metabolism, with key metabolites such as phenylalanine, chenodeoxycholic acid, and cholic acid identified as responsive markers [64]. Treatment with chitosan nanoparticles has been shown to regulate gut microbiota associated with oxidative stress, inflammation, and lipid deposition [65]. The results of this study are consistent with the well‐established properties of silymarin and chitosan.

Subsequently, the correlation analysis revealed that IL‐1β, TNF‐α, and IL‐6, along with liver function indicators AST and ALT, were negatively correlated with 3‐deoxy‐2‐keto‐6‐phosphogluconic acid and positively correlated with D‐ribose, D‐biotin, and creatinine, whereas the anti‐inflammatory factors IL‐10 and IL‐4 showed the opposite trend (Figure 9E). Additionally, the relative abundance of *Bacteroides*, *Fusobacterium*, and *Megamonas* was positively correlated with 3‐deoxy‐2‐keto‐6‐phosphogluconic acid levels, but negatively correlated with D‐ribose, D‐biotin, and creatinine (Figure 9F). These findings suggest that inflammatory and liver function markers are closely linked to specific metabolic pathways and gut microbiota profiles, highlighting the potential for therapeutic interventions targeting these interactions. The regulatory effect of Sil@PBACS Nps on fecal metabolites and gut microbiota may be related to pathways associated with sphingolipid metabolism and oxidative stress regulation. However, the precise causal relationship between specific microbes, metabolites, and pathways needs to be verified through targeted methods such as microbial intervention, metabolite supplementation, or pathway‐specific functional experiments. Another limitation of the present study is that intestinal tissues were not collected; therefore, direct evidence for barrier restoration, including tight junction protein expression and mucosal integrity, is lacking. Although the observed microbial and fecal metabolic changes were accompanied by improved hepatic outcomes, the role of the intestinal barrier in mediating this gut‐liver interaction remains to be directly verified.

### 2.11 Sil@PBACS Nps Reduce Post‐Hepatectomy Liver Pathology via Modulation of Inflammation and Lipid Metabolism

**FIGURE 10:**
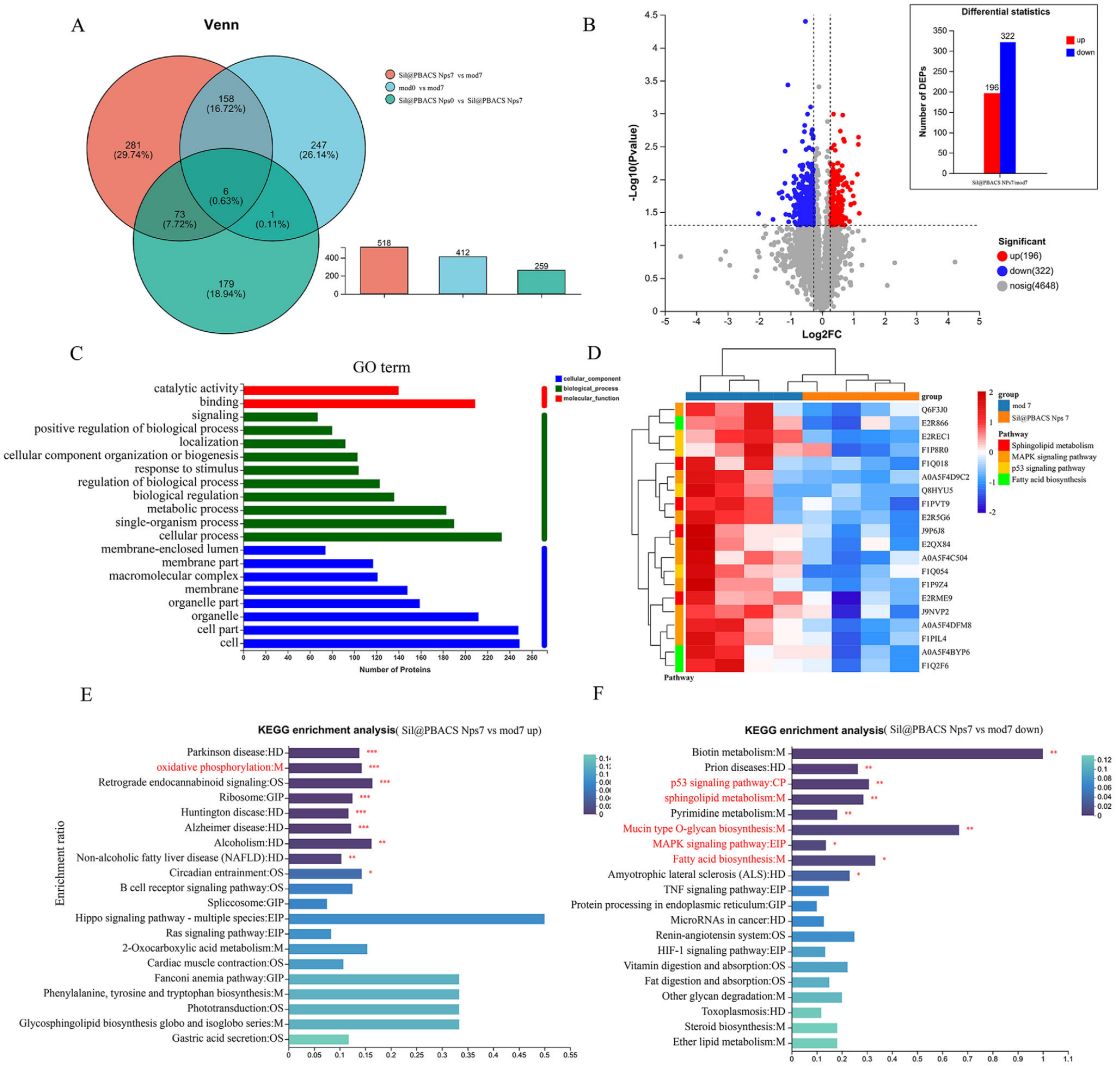
Proteomics analysis of liver tissue from hepatectomized dogs treated with Sil@PBACS Nps. (A) Venn diagram. (B) Volcano plot for whole identified proteins in Sil@PBACS Nps 7 vs mod7 (FC >1.2 or <0.83, *q* < 0.05). (C) GO enrichment analysis. (D) Heatmap of DEPs enriched in the four main pathways. KEGG enrichment analysis upregulated (E) and downregulated (F). (*p < 0.05, **p < 0.01, ***p < 0.001, ****p < 0.0001).

The liver plays a crucial role in sphingolipid metabolism, housing all identified sphingolipids and expressing the complete range of sphingolipid metabolic pathways [66]. To elucidate the mechanisms by which chitosan aids liver recovery post‐hepatectomy, we conducted proteomic analysis of liver samples from Sil@PBACS Nps and mod groups on day 7, compared to day 0. This analysis identified 4648 proteins or protein subunits, with 518 differentially expressed proteins (DEPs), including 322 downregulated and 196 upregulated (Figure 10A,B). Animals treated with Sil@PBACS Nps exhibited significant enrichment in biological processes related to cellular, single‐organism, and metabolic activities, particularly in catalytic activity, binding, and cellular component categories (Figure 10C).

Furthermore, the analysis revealed that all 20 DEPs associated with key signaling pathways‐sphingolipid metabolism, MAPK signaling, p53 signaling, and fatty acid biosynthesis were downregulated in the Sil@PBACS Nps group compared to mod (Figure 10D). KEGG pathway enrichment highlighted that upregulated DEPs were mainly involved in oxidative phosphorylation (Figure 10E), while downregulated DEPs were predominantly enriched in lipid metabolism‐related pathways, including sphingolipid metabolism and fatty acid biosynthesis (Figure 10F). Disruption of sphingolipid metabolism is a critical factor in hepatocyte death and is linked to various liver diseases and injuries [67]. A previous study has shown that enhancing sphingolipid levels in hepatocytes improved respiration under fatty‐acid overload [68, 69], suggesting that sphingolipid transfer to the liver might contribute to microbiota‐mediated liver function.

Previous studies have shown that p53 is crucial in regulating the cell cycle, DNA repair, apoptosis, and other biological functions, particularly under Sil@PBACS Nps treatment [70]. Similarly, maltol has demonstrated the potential to prevent liver damage in D‐gal‐induced aging mice by reducing oxidative stress through p53/p21/p16 and PI3K/Akt‐mediated pathways [71]. Furthermore, the MAPK pathway, a key cytokine‐associated signaling pathway, regulates various biological effects such as oxidative stress, inflammatory responses, apoptosis, proliferation, and differentiation, with chitosan administration shown to enhance its hepatoprotective effects [72]. Nutrient conversion to energy through respiration is critical, and defects in enzymes involved in oxidative phosphorylation can impair ATP production, leading to liver cell necrosis and apoptosis [73]. One study highlighted that the hepatoprotective effect of chlorogenic acids might be linked to enhanced ATP production, stimulation of mitochondrial oxidative phosphorylation, and inhibition of glycolysis [74]. Furthermore, proteomics research on emodin‐induced liver injury in rats indicated that the oxidative stress injury may result from mitochondrial dysfunction, affecting fatty acid β‐oxidation, the TCA cycle, and oxidative phosphorylation [75]. In summary, these research findings highlight changes in multiple key pathways (such as p53, MAPK, and oxidative phosphorylation), especially by regulating oxidative stress and energy metabolism, which may potentially alleviate liver damage.

### 2.12 Molecular Docking and Molecular Dynamics Simulation

**FIGURE 11:**
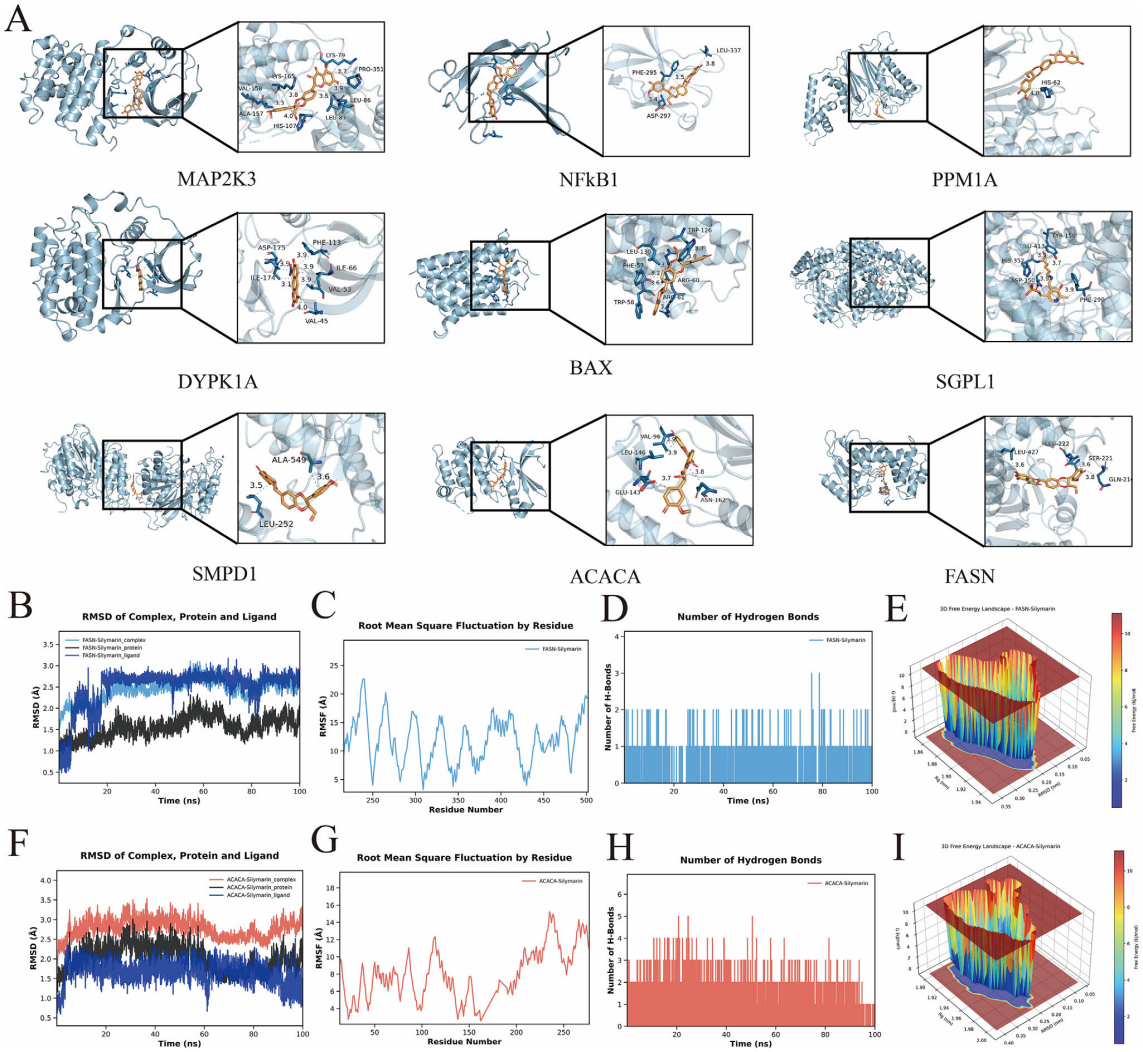
(A) The interaction and binding pattern of silymarin with core proteins related to sphingolipid metabolism and fatty acid biosynthesis. (B) RMSD results of silymarin targeting the FASN protein; (C) RMSF results; (D) changes in hydrogen bonding; (E) Gibbs free energy landscape; silymarin targeting the ACACA protein; (F) RMSD results; (G) RMSF results (H); changes in hydrogen bonding; (I) Gibbs free energy landscape.

The binding energies of the drug‐protein complexes are shown in Table S10. The interaction patterns and binding characteristics between silymarin and the nine proteins involved in the core of sphingolipid metabolism and fatty acid synthesis were described through molecular docking analysis (Figure 11A). Specific intermolecular forces, including hydrogen bonds, weak hydrogen bonds, hydrophobic interactions, cation‐π interactions, and π‐π stacking, were systematically identified. All predicted binding energies of silymarin‐target complexes were lower than −5.0 kilocalories per mole, indicating good and relatively strong binding affinity. It is notable that the two target proteins, acetyl‐CoA carboxylase α (ACACA) and fatty acid synthase (FASN), have been reported to be closely related to immune cell infiltration [76]. By down‐regulating the transcription factor SREBP1, they can reduce the expression of FASN and ACACA, thereby inhibiting the interaction between α‐tubulin and FASN, ACACA, and ACLY, and reducing the content of intracellular fatty acids and mitochondrial phospholipids; knockdown of FASN will decrease mitochondrial membrane potential, increase reactive oxygen species, mitochondrial abnormalities, and apoptosis [77]. Therefore, ACACA and FASN became the preferred candidate objects for our further research.

Subsequent molecular dynamics (MD) simulations were conducted for these two proteins. The root mean square deviation (RMSD) analysis showed that the FASN‐silymarin complex reached structural equilibrium after approximately 25 nanoseconds (Figure 11B). The root mean square fluctuation (RMSF) curve presented several distinct peaks (Figure 11C), indicating that the local flexibility of specific protein regions was enhanced during ligand binding. In the latter part of the simulation, the number of intermolecular hydrogen bonds between silymarin and FASN remained stable at three (Figure 11D), supporting a stable and persistent interaction [78]. The free energy landscape mapping based on principal component analysis (PCA) (Figure 11E) indicated that the main conformational ensemble of the FASN‐silymarin complex was concentrated within the range of 0.0 to 0.35 for PC1 values, reflecting a clear, low‐energy conformational basin. Similar analysis for the ACACA‐silymarin complex (Figure 11F–I) showed earlier RMSD convergence, higher average hydrogen bond numbers, and overall reduced conformational variability. Overall, this indicates that the binding stability of silymarin with key regulatory proteins in sphingolipid metabolism and fatty acid biosynthesis is higher. These computational analyses suggest that selected silymarin components may exhibit favorable interactions with the lipid metabolism‐related proteins FASN and ACACA under the modeled conditions. These findings are hypothesis‐generating and do not demonstrate direct target engagement or functional regulation in biological systems. Further biochemical and functional studies are required to validate these predicted interactions and determine their biological significance.

### 2.13 Integrated Multi‐Omics Data Clarify How Sil@PBACS Nps Reduce Liver Injury From Hepatectomy

**FIGURE 12:**
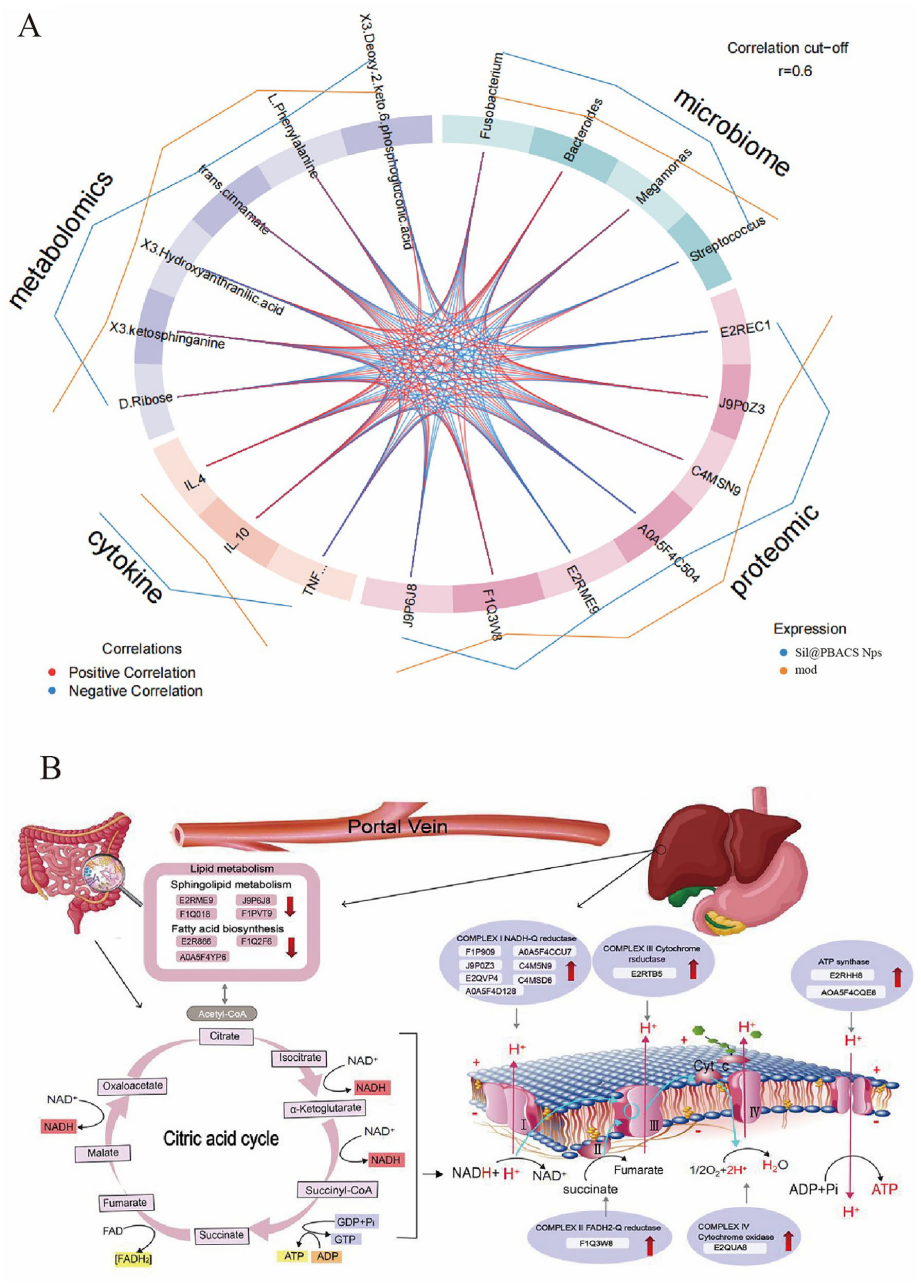
Correlation analysis between the microbiome, metabolome, proteome, and cytokines. (A) The Circos plot displays correlations between the microbiome, metabolome, proteome, and cytokines. Red and blue indicate positive and negative correlations (r > 0.6), respectively. (B) Schematic diagram of the relationship between liver proteins and gut microbiota response to liver injury in hepatectomized dogs after feeding Sil@PBACS Nps.

We utilized a random‐forest model to identify significantly expressed biomarkers with an MDA >4 (Figure S7), and the receiver operating characteristics (ROC) curve showed an AUC >0.7, complemented by the confusion matrix. DIABLO software was employed to examine correlations among the microbiome, metabolomics, and proteomics, with visualization of loading weights for each selected variable. Correlation Circos plots indicated that proteins F1Q3W8, C4MSN9, and J9P0Z3, involved in oxidative phosphorylation, positively correlated with *Bacteroides*, *Fusobacterium*, and *Megamonas*, as well as fecal metabolites 3‐ketosphinganine and 3‐hydroxyanthranilic acid, and anti‐inflammatory factors IL‐4 and IL‐10. Conversely, these proteins negatively correlated with the *Streptococcus* genus and the pro‐inflammatory factor TNF‐α (Figure 12A). This suggests that monitoring microbiota could be crucial for improving liver health, not only post‐hepatectomy but also across a range of liver diseases, warranting further exploration of potential pathway interactions.

Further analysis of key proteins enriched in the most altered pathways revealed that the oxidative phosphorylation pathway, featuring enzymes such as NADH reductase (e.g., F1P909 and J9P0Z3), cytochrome C reductase/oxidase (e.g., E2RTB5 and E2QUA8), and ATP synthase (e.g., E2RHH8 and A0A5F4CQE8), was significantly upregulated in the liver of Sil@PBACS Nps dogs *(p <0.05*, Figure 10B), indicating enhanced ATP generation. Interestingly, proteins involved in fatty acid biosynthesis (e.g., E2R866, F1Q2F6, and A0A5F4BYP6) were decreased in the liver of Sil@PBACS Nps‐treated dogs, while gut bacteria in these dogs showed enrichment in both fatty acid biosynthesis and the TCA cycle, which is associated with energy metabolism (Figure 12B).

These results indicate that Sil@ PBACS Nps regulate the microbiota along the intestinal‐liver axis, influencing potential liver effects, and upregulate the level of oxidative phosphorylation and regulate the fatty acid biosynthesis pathway. They can enhance liver energy metabolism after liver resection, providing a promising intervention strategy for improving liver health in the context of liver resection.

Although Sil@PBACS Nps showed robust hepatoprotective effects in the canine hepatectomy model, this large‐animal experiment was designed as a translational efficacy assessment of the optimized formulation rather than a definitive component‐level comparison. Because free silymarin and empty PBACS groups were not included in the canine study, the present data cannot unequivocally distinguish the individual contribution of silymarin, the PBACS carrier, or potential carrier–drug interactions in this model. The preliminary murine study supports the improved efficacy of Sil@PBACS Nps compared with free silymarin and PBACS alone, but future large‐animal studies with expanded control groups are required to confirm nanoparticle superiority and define the relative contribution of each component.

### 2.14 Coordinated Intestinal and Hepatic Protein Responses Associated With Sil@PBACS NP Treatment After Hepatectomy

**FIGURE 13:**
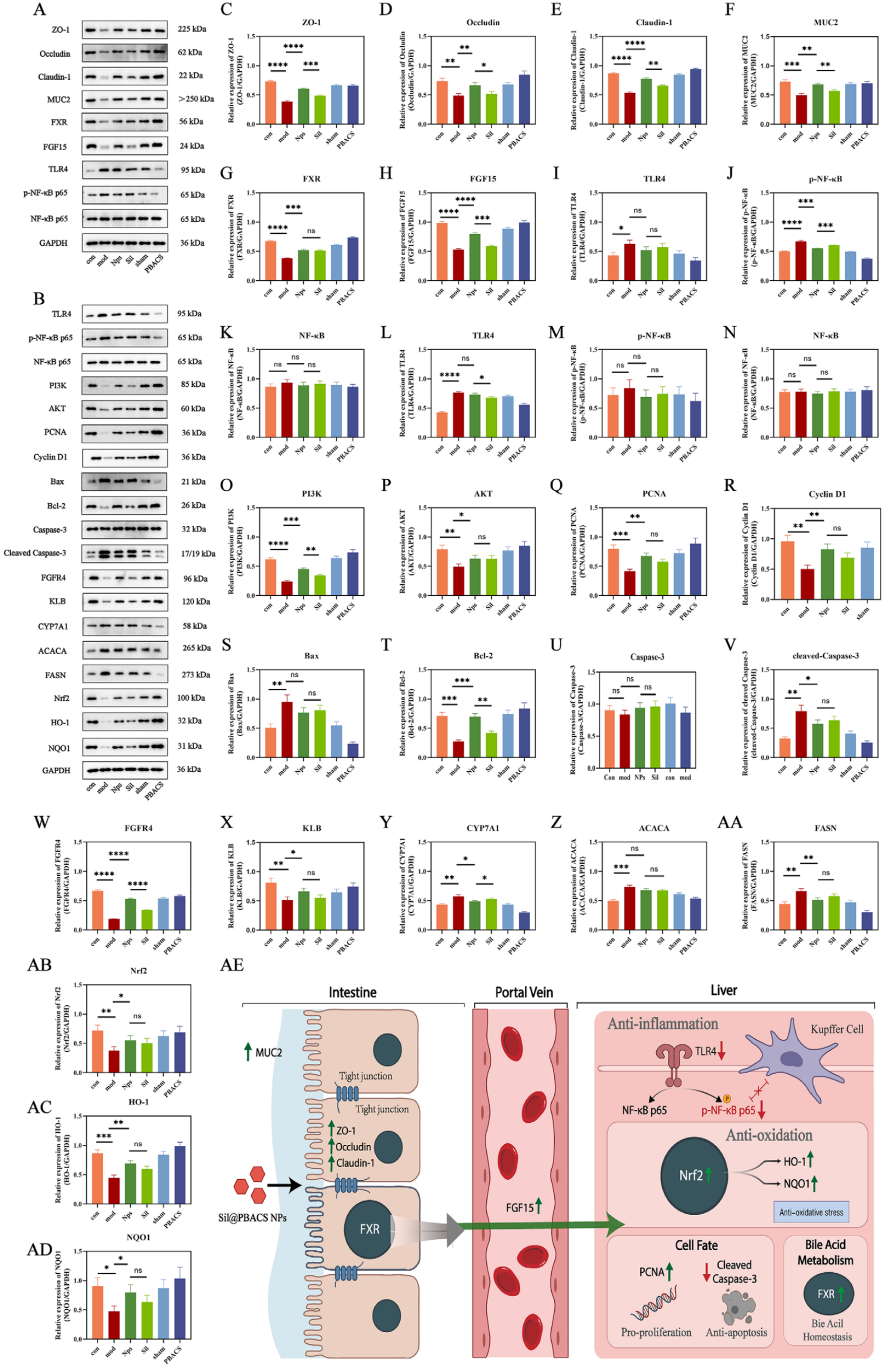
Western blot analysis of intestinal barrier function and hepatic gut–liver axis‐related proteins in mice. (A) Representative Western blot bands of intestinal barrier‐related proteins, FXR‐FGF15/19 pathway components, and TLR4/NF‐κB pathway components in mouse intestinal tissues. (B) Representative Western blot bands of TLR4/NF‐κB pathway components, proliferation markers, apoptosis‐related proteins, and antioxidant defense enzymes components in mouse hepatic tissues. (C–K) Quantitative analysis of mouse intestinal tissues normalized to GAPDH. (L‐AD)Quantitative analysis of mouse hepatic tissues normalized to GAPDH. (AE) Proposed underlying mechanisms of Sil@PBACS Nps‐mediated hepatic protection are linked to intestinal barrier restoration and gut‐liver axis‐regulated inflammatory, oxidative stress, regenerative, and apoptotic pathways. (con): without any treatment; (mod): hepatectomy model group; (Nps): hepatectomy + Sil@PBACS Nps group (Sil): hepatectomy + free silymarin group. Sham operation group (Sham): with incision of abdominal wall but suture without drug treatment and (PBACS): hepatectomy + PBACS group. Each lane in the blots represents a protein sample derived from an individual mouse, and the displayed blots are representative of these independent biological replicates. Band intensities were quantified via densitometry and normalized to GAPDH, which was used as the loading control. Statistical differences were determined using one‐way ANOVA with Bonferroni's multiple comparison test (Data are presented as mean ± SD; n = 3 biologically independent animals per group, *p < 0.05, **p < 0.01, ***p < 0.001, ****p < 0.0001).

To examine the intestinal and hepatic responses suggested by the multiomics analysis, selected proteins were assessed by Western blot in murine tissues. In colonic tissues, hepatectomy was associated with reduced expression of ZO‐1, Occludin, Claudin‐1, and MUC2. Compared with the hepatectomy model group, Sil@PBACS NP treatment increased the expression of these proteins involved in epithelial barrier structure and mucus production. FXR and FGF15 expression was also increased, whereas TLR4 was reduced. These findings support coordinated changes in intestinal bile acid signaling and inflammatory responses following treatment (Figure 13A,C–K).

In liver tissues, Sil@PBACS NP treatment reduced the expression of TLR4 and increased the expression of Nrf2, HO‐1, and NQO1. PCNA and Cyclin D1 expression was also increased, consistent with enhanced proliferative activity during hepatic recovery. In parallel, Bax expression was reduced, whereas Bcl‐2 expression was increased. These changes were associated with lower inflammatory and apoptotic signaling and stronger antioxidant and regenerative responses in the liver (Figure 13B, L‐AD). Canine intestinal tissues were not collected. Murine colonic tissues were available and provided protein‐level evidence for barrier‐associated responses; however, direct functional permeability assessment and intestinal validation in the canine model remain unavailable.

Together, these findings provide protein level evidence of coordinated intestinal and hepatic responses associated with Sil@PBACS NP treatment after hepatectomy. However, they do not establish a causal pathway involving the microbiota, microbial metabolites, the intestinal barrier, and the liver (Figure 13AE). Further studies involving microbiota manipulation, metabolite supplementation, and targeted pathway interventions are required to determine the contribution of individual components of the intestinal and hepatic response. Quantitative pharmacokinetic and biodistribution analyses, together with expanded canine studies that include free silymarin and blank PBACS Nps, would also help distinguish the respective contributions of the active compound, the carrier, and the complete formulation. Dose optimization, tolerance to repeated administration, long term safety, and validation in different hepatectomy models will be important for further translational evaluation.

## 3 Conclusions

In this study, Sil@PBACS Nps were prepared to serve as a multifunctional delivery system for silymarin. The optimized formulation exhibited uniform particle size, high encapsulation efficiency, good colloidal stability, and sustained drug release in vitro. Compared with free silymarin, Sil@PBACS Nps significantly improved antioxidant and antibacterial activities, likely due to enhanced solubility, controlled release, and the greater formulation efficacy of the chitosan matrix. The nanoparticles demonstrated favorable biocompatibility, exhibiting low cytotoxicity toward hepatic cells and excellent hemocompatibility. In the canine liver resection model, Sil@PBACS Nps effectively attenuate post‐hepatectomy liver injury, and multi‐omics evidence supported the existence of potential mechanistic associations between the liver function and the composition of the intestinal microbiota, and between liver function and key metabolic pathways. Notably, treatment with Sil@PBACS Nps increased the relative abundance of *Bacteroides*, *Fusobacterium*, and *Megamonas*, which might be associated with reduced inflammation and improved metabolic profiles. Multi‐omics analyses revealed changes in oxidative phosphorylation and modulation of fatty acid biosynthesis, as well as significant alterations in fecal metabolites linked to sphingolipid metabolism and the pentose phosphate pathway. While the present study demonstrates the therapeutic potential of Sil@PBACS Nps in a controlled experimental setting, it does not fully recapitulate the complexity of clinical conditions, which may involve heterogeneous disease states, comorbidities, and variable treatment responses. Overall, this study provides a robust foundation for the application of chitosan‐based nanocarriers in hepatoprotective efficacy and efficacy of hydrophobic phytochemicals such as silymarin, offering new avenues for therapy in hepatic disease management.

## 4 Materials and Methods

### 4.1 Reagents

Silymarin (Sil, Solarbio, ≥98%), sodium tripolyphosphate (TPP, Sigma, >85%), formic acid (Rhawn, chromatographic grade, ≥99%), methanol (Rhawn, chromatographic grade, ≥99.9%), anhydrous ethanol (Fuyu Reagents, AR), sodium hydroxide (NaOH, ≥96%) and glacial acetic acid (AR) were all purchased from the Beijing Chemical Factory. Chitosan with an average molecular weight of 3000 Da (purity≥99.3%, batch No. M‐KG‐2204001) was purchased from Zhejiang Golden Shell Pharmaceutical Co., Ltd. *Staphylococcus aureus* (ATCC 29740) and *Escherichia coli* (CVCC 1450) were purchased from the China General Microbiological Culture Collection Center (CGMCC) and the China Veterinary Culture Collection Center (CVCC), respectively.

### 4.2 Preparation of Phenylboronic Acid‐Modified Chitosan

Phenylboronic acid‐modified chitosan (PBACS) was synthesized via a carbodiimide‐mediated coupling reaction. Briefly, 3‐carboxyphenylboronic acid (PBA, 0.30 g, 2 mmol) was dissolved in 30 mL dimethyl sulfoxide (DMSO), followed by the addition of 1‐ethyl‐3‐(3‐dimethylaminopropyl) carbodiimide hydrochloride (EDC, 0.384 g, 2 mmol) and N‐hydroxysuccinimide (NHS, 0.230 g, 2 mmol). The solution was stirred at room temperature for 2 h to activate the carboxylic group of PBA. Separately, 0.20 g of low‐molecular‐weight chitosan (degree of deacetylation ≥85%) was fully dissolved in 20 mL of 1% (v/v) ice acetic acid, and the pH was adjusted to pH 5.5 using 1 mol/L NaOH. The activated PBA solution was then added dropwise to the chitosan solution under continuous stirring, maintaining a molar ratio of chitosan: PBA = 1:3. The reaction was allowed to proceed for 24 h at room temperature. After completion, the reaction mixture was transferred into a dialysis bag (MWCO = 3500 Da) and dialyzed against deionized water for 48 h, with water changes every 6 h to remove unreacted reagents and DMSO. The dialyzed solution was then lyophilized to obtain PBACS.

### 4.3 Characterization

FTIR analysis was conducted to verify the chemical conjugation of phenylboronic acid onto chitosan and to characterize the functional groups present in the Sil@PBACS Nps. FTIR spectra were acquired using a Nicolet iS5 spectrometer (Thermo Fisher Scientific, USA) over the range of 4000–400 cm^−^^1^, with a resolution of 4 cm^−^^1^ and 32 scans per sample. Key absorption bands were examined to confirm the presence of characteristic peaks associated with chitosan, silymarin, and the boronic acid moiety, as well as the formation of new bonds indicative of successful conjugation. Sil@PBACS Nps was placed onto a silicon wafer and dried at room temperature. The dried sample was then adhered to double‐sided conductive tape and gold‐coated for 2 min using a sputter coater. The SEM images of the Sil@PBACS Nps were obtained (JSM‐840, JEOL, Japan).

### 4.4 In Vitro Drug Release

To evaluate the in vitro release behavior of Sil@PBACS Nps, 20 mL of the nanoparticle suspension was placed in a dialysis bag and immersed in 100 mL of PBS buffer (pH 7.4) or PBS containing 1% (v/v) freshly prepared H_2_O_2_ (pH 7.4). The release medium was maintained at 37°C and stirred continuously at 150 rpm. At predetermined time points (0.25, 0.5, 0.75, 1, 2, 3, 4, 6, 8, 10, 12, 18, and 24 h), 1 mL of the external medium was withdrawn and replaced with an equal volume of fresh PBS at 37°C to maintain sink conditions. The concentration of silymarin in each sample was determined by HPLC. Furthermore, following the same procedure, the Sil@PBACS Nps were immersed in a pH 5.0 citric acid‐Na_2_HPO_4_ buffer solution and then tested to determine the drug release under acidic conditions. All experiments were performed in triplicate (n = 3). The release efficiency (Et) was calculated using the following formula:

$$\mathrm{Et}\left(\%\right)=\frac{P1}{P2}\times 100\%$$

Et(%) represents the release efficiency, P1 is the amount of Sil released from the dialysis bag, P2 is the total amount of Sil initially incorporated into the Sil@PBACS Nps.

### 4.5 In Vitro Antioxidant Assay

The ABTS^+^ storage solution was prepared according to the method described in previous studies [79]. Briefly, 38.4 mg/mL of ABTS was dissolved in 10 mL of water to obtain solution A, and 13.4 mg/mL of potassium persulfate was dissolved in 10 mL of water to obtain solution B. Equal volumes of solutions A and B were mixed and stored at room temperature in the dark for 12 h to yield the ABTS^+^ storage solution. Prior to use, the ABTS^+^ storage solution was diluted with deionized water to adjust the absorbance at 734 nm to 0.7 ± 0.02, which resulted in the ABTS^+^ working solution.

For the assay, 0.2 mL of the sample at varying concentrations (2, 1, 0.5, 0.25, 0.125, 0.0625 mg/mL) was added to a 15 mL centrifuge tube, followed by the addition of 3.8 mL of the ABTS^+^ working solution. After thorough mixing, the reaction was allowed to proceed for 6 min, and the absorbance was measured at 734 nm.

Following the method described in a previous study [80], DPPH was dissolved in methanol at a concentration of 0.4 mmol/L and protected from light. A 0.2 mL aliquot of the DPPH solution was added to a 96‐well plate, followed by 0.05 mL of the sample at various concentrations (2, 1.5, 1, 0.5, 0.25, 0.125, 0.0625 mg/mL). The mixture was allowed to react in the dark at room temperature for 30 min, after which the absorbance was measured at 517 nm.

### 4.6 In Vitro Antibacterial Assay

The antimicrobial activity of the samples was assessed using the micro‐broth dilution method. Under sterile conditions, a sterilized sample (5 mg/mL) was serially diluted in sterile water within a 96‐well plate. Each well contained 100 µL of the serially diluted sample and 100 µL of bacterial suspension at a concentration of 1.5 × 10^5^ CFU/mL, resulting in a range of sample concentrations. Positive control wells contained 100 µL of bacterial suspension and 100 µL of broth, while negative control wells contained 200 µL of broth alone.

Following incubation at 37°C for 12 h, 100 µL of the suspension from the well with the lowest concentration showing no visible turbidity or sediment was plated onto solid media. If the colony count on the solid media was ≤5, this concentration was designated as the minimum inhibitory concentration (MIC). If more than 5 colonies were present, the next higher concentration from the serial dilution was plated. The minimal bactericidal concentration (MBC) was recorded as the lowest concentration at which no colonies grew on the media.

The antibacterial activity of Sil@PBACS Nps was evaluated by measuring the inhibition zones against *S. aureus* and *E. coli*. Petri dishes containing a bacterial suspension at a concentration of 10^5^ CFU/mL were prepared. Blank control (1% DMSO), free silymarin (Sil), PBACS, and Sil@PBACS Nps were introduced into separate dishes. The concentration of free silymarin matched that of the Sil encapsulated in the Sil@PBACS Nps. The plates were incubated at 37°C for 24 h, after which the diameters of the inhibition zones surrounding each sample were measured using a vernier caliper. Each experiment was conducted in triplicate to ensure reproducibility.

After culturing the bacteria to the logarithmic growth phase, they were diluted and mixed with each group. The bacterial solution was incubated at 37°C for 12 h, then diluted 1000 times and inoculated onto LB agar plates. After incubation for 24 h, colony images were taken and colony counts were performed using ImageJ software. Furthermore, the viability/deadness cell staining method was used to evaluate the antibacterial performance. After each treatment, 100 µL of the bacterial solution was transferred to a 96‐well plate, and 1 µL of the mixed dye solution (DMAO/PI) was added to each well. The plates were incubated at room temperature in the dark for 15 min. The bacterial activity was observed using an upright fluorescence microscope (OLYMPUS BX51): live bacteria were stained green by DMAO, while dead and damaged cells were stained red by PI. The degree of bacterial survival and death was evaluated through image analysis.

### 4.7 Hemolytic Activity Test

The hemolytic activity of Sil@PBACS Nps was evaluated using rabbit red blood cells (RBCs). Fresh RBCs were washed three times with phosphate‐buffered saline (PBS) by centrifugation at 1000 × g for 15 min until the supernatant became clear. The washed RBCs were then resuspended in PBS to obtain a 4% (v/v) erythrocyte suspension. A total of 100 µL of this suspension was mixed with 100 µL of Sil@PBACS Nps at various concentrations in 96‐well plates and incubated at 37°C for 1 h. After incubation, the mixtures were centrifuged at 1000 × g for 15 min, and the absorbance of the supernatant was measured at 540 nm. PBS treated RBCs served as the negative control (0% hemolysis), while RBCs treated with 0.1% (v/v) Triton X‐100 served as the positive control (100% hemolysis). The hemolysis percentage was calculated using the following equation:

$$\mathrm{Hemolysis}(\%)=\frac{OD_{540}\;\mathrm{sample}-OD_{540}\mathrm{PBS}}{OD_{540}\;100\%\;\mathrm{hemolysis}-OD_{540}\mathrm{PBS}}\;\times 100\%$$

### 4.8 Cell Viability Assay

The cytotoxicity of Sil@PBACS Nps toward AML‐12 (murine hepatocytes) and HepG2 (human hepatocellular carcinoma cells) cells was evaluated using the Cell Counting Kit‐8 (CCK‐8, Dojindo, Japan) assay. Briefly, cells were seeded in 96‐well plates at a density of approximately 4 × 10^3^ cells per well and cultured at 37°C in a humidified incubator with 5% CO_2_ for 24 h to allow for cell adherence. The culture medium was then replaced with 100 µL fresh medium containing various concentrations of Sil@PBACS Nps. After 24 h of incubation, 10 µL of CCK‐8 reagent was added to each well and incubated for an additional 2 h under the same conditions. The optical density (OD) at 450 nm was measured using a multimode microplate reader (BioTek, USA). Cell viability was calculated as a percentage relative to the untreated control group. Each experiment was conducted in triplicate to ensure reproducibility.

### 4.9 Animals and Hepatectomy

Canine animal model: Twelve healthy adult female beagle dogs (7–8 months old, 13.2 ± 3.1 kg) were obtained from Beijing Marshall Biotechnology Co., Ltd., China (No. SYXK2023‐0047). The experimental protocol was approved by the Animal Ethics Committee of Beijing University of Agriculture (BUA812403013). After 2 weeks of acclimatization on a standard diet, the dogs were randomly divided into the model group (mod, n = 6) and the Sil@PBACS Nps group (n = 6). Both groups were fed the normal diet twice daily, while the Sil@PBACS Nps group additionally received Sil@PBACS Nps at 80 mg/kg for 7 days before laparoscopic hepatectomy and for 28 days thereafter. Laparoscopic hepatectomy was performed according to our previously established protocol [81, 82], with minor modifications. Briefly, the dogs were anesthetized with isoflurane inhalation and placed in the supine position. After pneumoperitoneum establishment and trocar placement, the left hepatic lobe, including the left lateral and left medial lobes, was removed to establish a partial hepatectomy model. The resection extent was approximately 40% of the total liver volume according to the previously reported protocol [81, 82]. Postoperative care, perioperative monitoring, and model validation criteria were also performed as described previously [81, 82]. Fecal samples were collected on days 0, 7, and 28 and stored in liquid nitrogen for 16S rRNA gene sequencing and metabolomics analysis. Blood samples were collected on days 1, 3, and 7, allowed to clot, centrifuged at 3000 × g for 10 min, and stored at −80°C for further analysis. Liver tissue samples were collected during laparoscopy on days 0, 7, and 28 for proteomic analysis and histopathological examination.

Mouse animal model: Female SPF‐grade Kunming mice (6–7 weeks old, 20–25 g) were obtained from Sibefu (Beijing) Biotechnology Co., Ltd and acclimated for 1 week prior to experimentation. The experimental protocol was approved by the Animal Ethics Committee of Beijing University of Agriculture (BUA812403014). All procedures were approved by the Animal Ethics Committee of Beijing Agricultural University. In order to systematically compare the effects of each component of the preparation, mice were randomly divided into six groups: control group (con), sham operation group (sham), hepatectomy model group (mod), hepatectomy + PBACS group (PBACS), hepatectomy + free silymarin group (Sil) and hepatectomy + Sil @ PBACS Nps group (Nps), with six mice in each group (n = 6). Treatments were administered by oral gavage for 7 consecutive days before surgery, while the sham group received saline. Partial hepatectomy was performed by resection of the left lateral hepatic lobe using a procedure analogous to that applied in the canine experiment. On postoperative day 7, blood was collected, allowed to clot, and centrifuged at 3000 × g for 10 min. Serum samples were stored at −80°C, and liver and intestinal tissues were collected for subsequent analyses.

### 4.10 In Vivo Fluorescence Imaging of Cy5.5‐Labelled Sil@PBACS Nps

To evaluate the local distribution and retention of Sil@PBACS Nps, Cy5.5‐labelled Sil@PBACS Nps were prepared for in vivo fluorescence imaging. Mice were randomly divided into Cy5.5‐labelled PBS and Cy5.5‐labelled Sil@PBACS Nps group (n = 3 per group). Drugs were administered by gavage. At predetermined time points of 1, 6, 12, 24 after administration, mice were anesthetized and imaged using an AniView in vivo fluorescence imaging system. Fluorescence images were acquired using the Cy5.5 channel with excitation/emission wavelengths of 675/720 nm. Fluorescence signals in the mammary gland region were recorded to evaluate local retention of the nanoparticle formulation. Image acquisition and analysis were performed using AniView and BioAnaly/BLTBioAnaly software. The Cy5.5‐labelled PBS group was used as the fluorescence control to compare retention of the nanoparticle formulation.

### 4.11 Biochemical Analysis

The liver function markers ALT (alanine aminotransferase) and AST (aspartate aminotransferase) were determined with a biochemical analyzer (Mindray Biomedical Electronics Co., Ltd., Shenzhen, China). The cytokines, tumor necrosis factor‐α (TNF‐α), interleukin‐1β (IL‐1β), interleukin‐6 (IL‐6), interleukin‐10 (IL‐10), and interleukin‐4 (IL‐4) in serum were measured using enzyme‐linked immunosorbent assay (ELISA) kits (Huaying Biological Technology Co., Ltd, Beijing, China).

### 4.12 High‐Throughput Sequencing Analysis of Fecal Microbiota

Fecal microbial composition was analyzed by high‐throughput sequencing of the V3–V4 region of the bacterial 16S rRNA gene. Total genomic DNA was extracted from fecal samples using the AxyPrep DNA Gel Extraction Kit (Axygen Biosciences, Union City, CA, USA) according to the manufacturer's protocol [83, 84]. The V3–V4 region was amplified using primers 338F (5′‐ACTCCTACGGGAGGCAGCAG‐3′) and 806R (5′‐GGACTACHVGGGTWTCTAAT‐3′). Amplicon sequencing was performed on the Illumina MiSeq PE300 platform (Illumina, San Diego, CA, USA) by Majorbio Bio‐Pharm Technology Co., Ltd. (Shanghai, China).

Raw paired‐end reads were quality‐filtered, merged, and denoised according to the Majorbio standard pipeline. Low‐quality reads, ambiguous bases, and chimeric sequences were removed before downstream analysis. Taxonomic assignment was performed against the SILVA bacterial 16S rRNA database. Alpha‐ and beta‐diversity analyses, taxonomic composition analysis, and differential abundance analysis were performed using the Majorbio Cloud platform and R software. PICRUSt2 was used to predict potential KEGG functional profiles from the 16S rRNA sequencing data. These predictions were interpreted as exploratory functional inference rather than direct metagenomic evidence. All raw sequence data were deposited in NCBI under accession number **PRJNA991635**.

### 4.13 Metabolomics

Fecal metabolite profiles were analyzed using an ultra‐high‐performance liquid chromatography system coupled to a Thermo Q Exactive HFX mass spectrometer. Samples were separated on an Acquity UPLC HSS T3 column (Waters Corporation, USA), and data were acquired in both positive and negative ion modes. The mass range was set from 100 to 1000 m/z, and samples were maintained at 4°C during analysis.

Quality‐control samples were prepared by pooling aliquots from all samples and were injected periodically throughout the analytical sequence to monitor instrument stability and repeatability. Raw LC–MS data were converted to mzXML format using ProteoWizard and processed using an R‐based workflow developed by Biotree Biotech Co., Ltd. (Shanghai, China), including peak detection, extraction, alignment, integration, and normalization. Features with excessive missing values or poor reproducibility in QC samples were removed. Remaining missing values were imputed using the minimum value or half‐minimum value within each metabolite feature before statistical analysis.

Multivariate analysis was performed after data normalization. Differential metabolites were screened using variable importance in projection (VIP) > 1 and *p* < 0.05. Where applicable, multiple testing correction was performed, and adjusted p values were used to support pathway‐level interpretation. The metabolomics results were interpreted together with microbiome, biochemical, and proteomic findings rather than as independent causal evidence. All raw sequence data were deposited in metaboLights under accession number **MTBLS15293**

### 4.14 Proteomics Analysis

Liver tissue samples were lysed, and protein concentrations were determined using a BCA assay kit (Thermo Fisher Scientific, USA). Equal amounts of protein from each sample were subjected to reduction, alkylation, enzymatic digestion, and tandem mass tag (TMT) labeling. The labeled peptides were reconstituted in UPLC loading buffer containing 2% acetonitrile, with ammonia used to adjust the pH to 10. High‐pH reversed‐phase peptide fractionation was performed using a C18 column (Waters, USA). The fractions were lyophilized and analyzed on a Q‐Exactive mass spectrometer (Thermo Fisher Scientific, USA). Full MS scans were acquired over a mass range of 350–1500 m/z, and the 15 most intense precursor ions were selected for MS/MS fragmentation. Raw Q‐Exactive data were searched using Proteome Discoverer software version 2.4 (Thermo Fisher Scientific, USA) against the corresponding protein database. Protein abundance data were normalized before statistical analysis. Missing values were filtered or imputed according to data completeness criteria. Differentially expressed proteins were identified using a two‐tailed Student's t‐test followed by Benjamini–Hochberg false‐discovery‐rate correction. Proteins with fold change > 1.2 or < 0.83 and BH‐FDR‐adjusted p‐value (q‐value) < 0.05 were considered differentially expressed.

Principal component analysis, volcano plots, and hierarchical clustering were performed using R software. Functional annotation and pathway enrichment analyses of differentially expressed proteins were performed using the Gene Ontology database and Kyoto Encyclopedia of Genes and Genomes database through the Majorbio Cloud platform. The proteomics results were interpreted in combination with fecal microbiota, fecal metabolomics, biochemical indices, and targeted protein validation. The analysis data has been uploaded to the CNCB‐OMIX database with the accession number **OMIX019484**.

### 4.15 Molecular Docking Analysis of Core Target Proteins

Molecular docking was performed to investigate the binding modes and affinities between silymarin and the key target proteins. Molecular docking was performed using AutoDock Vina, which calculated the binding energy (kcal/mol) between Sil and the proteins. Finally, the docking results were visualized using PyMOL software to generate detailed binding mode diagrams

### 4.16 Molecular Dynamics Simulations of Protein‐Sil Complexes

MD simulations were conducted on the FASN‐Sil and ACACA‐Sil complexes using Gromacs 2022.3 software. The small molecule (Sil) was preprocessed using AmberTools22, applying the GAFF force field and adding hydrogen atoms. The restricted electrostatic potential charges for the small molecule were calculated using Gaussian 16 W. The protein‐ligand system was modeled using the Amber99sb‐ildn force field. The system underwent energy minimization using the steepest descent algorithm, ensuring that any steric clashes or unrealistic geometries were resolved before further simulation. The equilibration process was then performed in two phases: 100 000 steps of NVT (canonical ensemble) equilibration and 100 000 steps of NPT (isothermal‐isobaric ensemble) equilibration. Each phase was conducted with a coupling constant of 0.1 ps and a duration of 100 ps. Finally, MD simulation was performed using a 2 fs time step over a total duration of 100 ns. Upon completion of the MD simulation, key parameters of the complex were computed using the built‐in GROMACS tool, including root mean square deviation (RMSD), root mean square fluctuation (RMSF), hydrogen bond interactions, and the Gibbs free energy landscape. Additionally, the binding free energy of the complexes was computed using the Molecular Mechanics/Poisson‐Boltzmann Surface Area (MM/PBSA) method.

### 4.17 Protein Extraction and Western Blot

Murine intestinal and liver tissues were collected. Tissue samples were washed with ice‐cold PBS and lysed in RIPA buffer containing protease and phosphatase inhibitors on ice for 30 min. The lysates were centrifuged at 14 000 × g for 15 min at 4°C, and the supernatants were collected for protein analysis. Total protein concentration was determined using a BCA protein assay kit (Beyotime Biotechnology, Shanghai, China) according to the manufacturer's instructions. Briefly, a standard curve was established, and the absorbance of each protein sample was measured to calculate protein concentration. Protein extracts were mixed with loading buffer (Smartbuffer) and denatured at 100°C for 15 min. Equal amounts of protein were separated by 10% SDS‐PAGE and electro‐transferred onto PVDF membranes (Beyotime Biotechnology, Shanghai, China).

The membranes were blocked with 5% non‐fat milk in TBST for 1 h at room temperature and incubated overnight at 4°C with the corresponding primary antibodies. For murine intestinal tissues, antibodies against ZO‐1, Occludin, Claudin‐1, FXR, FGF15/19, MUC2, TLR4, p‐NF‐κB p65, NF‐κB p65, and GAPDH were used. For murine liver tissues, antibodies against TLR4, p‐NF‐κB p65, NF‐κB p65, FGFR4, β‐Klotho/KLB, CYP7A1, PCNA, Cyclin D1, BAX, Bcl‐2, ACACA, FASN, Caspase‐3, Cleaved Caspase‐3, PI3K, Akt, Nrf2, HO‐1, NQO1, and GAPDH were used. All primary antibodies were purchased from Beyotime Biotechnology (Shanghai, China). After washing with TBST, the membranes were incubated with the corresponding HRP‐conjugated secondary antibodies for 1 h at room temperature. Immunoreactive bands were visualized using an enhanced chemiluminescence solution, and images were captured using a chemiluminescence imaging system. Band intensity was quantified using ImageJ software. Protein expression was normalized to GAPDH. Data are presented as mean ± SD″. n = 3 biologically independent animals per group.

### 4.18 Bioinformatics and Statistical Analysis

All data are presented as mean ± standard deviation (SD) from at least three independent experiments (n ≥3). Statistical significance was determined using one‐way analysis of variance (ANOVA), with p‐values less than 0.05 considered statistically significant. For the longitudinal biochemical data collected from the same animals at different postoperative time points, repeated‐measures ANOVA analysis was used. Graphs were generated using GraphPad Prism 8.0 and Origin 2021. Correlation analyses were performed to explore associations between gut microbiota, host metabolites, and clinical indicators. Specifically, Spearman correlation analysis was used to link microbial taxa with metabolites and inflammatory markers, while Pearson correlation analysis was applied to assess relationships between metabolites and clinical parameters. Random forest analysis was conducted using the “randomForest” package in R, with gut microbiota, fecal metabolites, differential proteins, and inflammatory indicators as input variables. The UC‐RF algorithm was employed to identify the most predictive features based on the maximum area under the curve (AUC).

## Author Contributions

**Jinjin Tong and Chaochao Luo**: writing – review & editing, investigation, supervision. **Zezheng Liu**: writing – original draft, writing – review & editing, visualization, software. **Xinyan Zhang**: investigation, data curation, resources. **Daixu Jia and Jiangfang Wang**: visualization and methodology. **Yang Sun and Haoran Shi**: visualization, validation. **Zhaonan Zhang**: validation and methodology. **Hua Zhang**: funding acquisition, methodology, project administration.

## Funding

This study was supported by the National Natural Science Foundation of China (Grant Nos. 32373086 and 32272904). The High‐Level Talent Research Initiation Program of Shihezi University (RCZK202364), and the Tianchi Talent Introduction Program of Xinjiang Uygur Autonomous Region (CZ001633).

## Conflicts of Interest

The authors declare no conflicts of interest.

## Supporting information




**Supporting File**: advs77847‐sup‐0001‐SuppMat.docx.

## Data Availability

The data that support the findings of this study are available from the corresponding author upon reasonable request.
