# Supplementary material for: ROS/pH‐Responsive Phenylboronic Acid–Chitosan Nanoparticles Improve Silymarin‐Mediated Hepatoprotection After Hepatectomy With Gut‐Liver Axis‐Associated Responses
Source: Adv Sci (Weinh). 2026 Sep 27:e77847. Online ahead of print. doi: 10.1002/advs.77847 (PMC13616251; doi:10.1002/advs.77847)
Supplement: Supplementary file 1 [file ADVS-9999-e77847-s001.docx]

**Supplementary Information**

**ROS/pH-Responsive Phenylboronic Acid–Chitosan Nanoparticles Improve Silymarin-Mediated Hepatoprotection after Hepatectomy with Gut-Liver Axis-Associated Responses**

**1 PBACS preparation method**

**1.1 Chitosan (CS) standard curve**

**Figure S1.** Standard curve for chitosan. CS was dissolved in 1% glacial acetic acid to obtain a standard stock solution of 4.00 mg/mL. The stock solution was diluted with 1% glacial acetic acid to obtain standard solutions with mass concentrations of 4.00, 3.00, 2.00, 1.00 and 0.50 mg/mL. Standard solutions of known concentrations were reacted with 10 mg/mL ninhydrin solution, the absorbance at 570 nm was measured, and a standard curve was prepared by linear regression analysis.

**1.2 Grafting rate of phenylboronic acid-chitosan (PBACS)**

The grafting rate of PBACS was measured by ninhydrin reaction. Standard solutions of chitosan at 4.00, 3.00, 2.00, 1.00 and 0.50 mg/mL were prepared. The standards were reacted with 10 mg/mL ninhydrin solution, the absorbance was measured at 570 nm, and the standard curve was drawn. The absorbance of the sample was calculated by reference to the standard curve.

**1.3 Single-factor experiment**

Four factors--the amount of activation solvent DMSO (10-60 mL), the molar ratio of PBA to CS (1:2 to 5:1) , the pH of the reaction system (pH 3-11) and the activation time (0.5 to 6 h)--were selected for individual testing. During each test, all other reaction conditions were kept constant. The orthogonal test of the four factors at three levels was carried out based on the results of the single-factor tests.

**Figure S2.** The influence of different factors on the grafting rate of PBA to CS. (A) amount of DMSO; (B) pH; (C) Ratio of PBA to CS; and (D) activation time. (n = 3).

**1.4 Orthogonal tests**

According to the results of the single factor experiment, the pH of the reaction system, the molar ratio of CS to PBA, the amount of solvent DMSO and the activation time had different effects on the grafting rate of PBA-CS. Therefore, independent orthogonal tests of the four factors at three levels were carried out: the pH of the reaction system (7,9, or 11), the molar ratio of PBA to CS of (5:1, 4:1, or 3:1), the amount of solvent DMSO of (30, 40, or 50 mL), and the activation time of 2, 4, or 6 h. The numbers under each factor in the table represent the 1^st^, 2^nd^, and 3^rd^ level of that factor. The orthogonal test was carried out with the highest grafting rate as the selection criterion.

According to the orthogonal results, the degree of influence of each factor on the grafting rate can be arranged from most to least (Table S1): C ( DMSO dosage ) > A (pH value)> D(activation time )> B (molar ratio). The *k* value in the table represents the positive influence degree of the three levels of the factor on the grafting rate. Based on these values, the preparation scheme with the highest grafting rate is A2B2C3D1 (pH of 9, 4:1 molar ratio of PBA to CS, 0.3 g of PBA, 50 mL of DMSO, and an activation time of 2 h). The grafting rate was determined by the ninhydrin chromogenic method used in the single factor test, and the grafting rate of PBA-CS obtained under the optimal preparation conditions was determined to be 58.6 ± 2.1 %.

**Table S1.** Orthogonal test results.

| Number | A (pH) | B (PBA:CS) | C (DMSO) | D (time) | Grafting rate (%) |
| --- | --- | --- | --- | --- | --- |
| 1 | 1 | 1∶1 | 1 | 1 | 17.55 % |
| 2 | 1 | 2∶1 | 3 | 2 | 41.72 % |
| 3 | 1 | 3:1 | 2 | 3 | 16.93 % |
| 4 | 2 | 1∶1 | 3 | 3 | 53.20 % |
| 5 | 2 | 2∶1 | 2 | 1 | 43.72 % |
| 6 | 2 | 3:1 | 1 | 2 | 15.31 % |
| 7 | 3 | 1∶1 | 2 | 2 | 25.07 % |
| 8 | 3 | 2∶1 | 1 | 3 | 24.07 % |
| 9 | 3 | 3:1 | 3 | 1 | 52.24 % |
| k1 | 0.2540224 | 0.3194143 | 0.1897784 | 0.3783717 |  |
| k2 | 0.3741194 | 0.3650416 | 0.2857421 | 0.2736748 |  |
| k3 | 0.3379175 | 0.2816034 | 0.4905387 | 0.3140127 |  |
| Range | 0.1200969 | 0.0834381 | 0.3007603 | 0.1046968 |  |

**1.5 Proton nuclear magnetic resonance**

The proton nuclear magnetic resonance spectrum shows the chemical shift of protons, which is consistent with the expected chemical structure. The broad peak at 3.6 ppm is attributed to the protons (1-6) on the C1-C6 positions of the chitosan units, while the chemical shifts related to the four aromatic protons (7-10) on the benzene ring of the PBA units are between 7.4 - 8.3 ppm, which is attributed to the proton resonance of the benzene ring in the PBA units. This indicates that the PBA has successfully grafted onto the chitosan backbone.

**Figure S3.** PBA-CS proton NMR spectrum.

**1.6 X-ray diffraction analysis**

In the diffraction pattern of chitosan, the sharp diffraction peak near 20° corresponds to the crystal structure of chitosan, indicating that there are a large number of crystalline regions formed by hydrogen bonds in chitosan molecules. In contrast, the diffraction peak intensity of PBA-CS between 20° and 30° was significantly reduced and showed a wide absorption peak. This change shows that after the grafting of phenylboronic acid, the crystallinity of chitosan is reduced, the hydrogen bonding force between molecules is weakened, and the original crystal structure is destroyed.

**Figure S4** X-ray diffraction comparison of PBA-CS with CS.

**2. Silymarin detection method**

**2.1 Construction of the silybin standard curve**

Silybin standards were dissolved in methanol to prepare a 300 μg/mL stock solution. The stock solution was then serially diluted with methanol to obtain concentrations of 300, 150, 100, 75, 60, and 30 μg/mL. After filtering the samples through a 0.22 μm membrane, they were analyzed by high-performance liquid chromatography (HPLC). A standard curve was generated by plotting the concentration of the standard solution on the x-axis and the corresponding peak area on the y-axis. The regression equation was calculated based on these values: Y = 23,950X + 29,140 with a correlation coefficient, R^2^ = 0.9997, demonstrating excellent linearity. This standard curve served as the reference for subsequent quantitative analyses. Using the peak area of silymarin and the silybin standard curve, the silybin content in the prepared silymarin nanoparticles was calculated to be approximately 29.73%. The retention times of the two silybin isomers were 6.42 min and 7.15 min, respectively.

**2.2 Determination of silybin content in Sil@PBACS Nps**

To determine the silybin content in the Sil@PBACS Nps, the silybin used in the nanoparticle preparation was dissolved in methanol to a concentration of 300 μg/mL. The samples were analyzed under identical chromatographic conditions as the standard solutions. The Sil@PBACS Nps were first centrifuged at 15,000 rpm for 30 minutes. The supernatants were collected and centrifuged again under the same conditions until no Tyndall effect was observed. The sample was then filtered through a 0.22 μm membrane and separated by HPLC (2695, Waters, USA) on a Diamonsil C18 column (5 μm, 4.6 mm × 150 mm). The mobile phase consisted of an aqueous phase of DIW with 0.1% formic acid and an organic phase of methanol. The injection volume was 10 μL, the flow rate was 1.0 mL/min, and detection was carried out at 287 nm.

The encapsulation efficiency and drug loading of the nanoparticles were evaluated using ultrafiltration-centrifugation. A 1 mL aliquot of the Sil@PBACS Nps suspension was accurately transferred into a 1.5 mL centrifuge tube and centrifuged at 15,000 rpm for 30 minutes at room temperature. After centrifugation, the supernatant was filtered through a 0.22 µm membrane, and the silymarin content in the supernatant was determined by HPLC at a wavelength of 254 nm. The encapsulation efficiency was calculated using the following formula (Zhang et al.2018):

Encapsulation Efficiency = $\frac{M1-M2}{M1}$ × 100 %

Where:

- M1 is the initial amount of silymarin,
- M2 is the amount of free silymarin in the filtrate.

The drug loading was calculated using the following formula (Zhao et al.2023):

Drug Loading = $\frac{M1-M2}{Mt}$ × 100 %

Where:

- M1 is the initial amount of silymarin,
- M2 is the amount of free silymarin in the filtrate,
- Mt is the total mass of the materials used to prepare the PBACS@Sil Nps.

**2.3 Single-factor experiments**

The influence of formulation and processing parameters on the characteristics of Sil@PBACS Nps was systematically evaluated, including encapsulation efficiency, drug loading, particle size, and PDI (Figure S5). Each parameter was investigated under controlled conditions, and changes in nanoparticle properties were analyzed to identify optimal preparation conditions.

****(A) TPP concentration.**** With a PBACS concentration of 1 mg/mL, a PBACS:Sil ratio of 2:1, and a stirring time of 30 min (all three parameters fixed), TPP concentrations ranging from 0.5 to 3 mg/mL were evaluated (Figure S5). Encapsulation efficiency and drug loading initially declined, reaching minimum values at 2.5 mg/mL, but increased sharply at 3 mg/mL, likely due to excess TPP destabilizing the system and promoting drug precipitation. The smallest particle size (174.3 ± 5.4 nm) and highest PDI (0.169 ± 0.021) were observed at 3 mg/mL, suggesting the coexistence of nanoparticles and free hydrophobic drug aggregates. These findings are consistent with prior reports (Chen et al.2024); a TPP concentration of 0.5–1.5 mg/mL was identified as optimal.

****(B) PBACS:TPP mass ratio.**** Keeping PBACS concentration at 1 mg/mL, the PBACS:TPP ratio was varied from 15:1 to 40:1 (Figure S5). A ratio of 30:1 resulted in the lowest encapsulation efficiency (15.91 ± 0.03%) and drug loading (2.33 ± 0.01%), possibly due to suboptimal crosslinking. In contrast, 40:1 achieved the highest encapsulation, but may have been affected by hydrophobic aggregation. A 20:1 ratio provided a balance between encapsulation efficiency and physical stability. ****(C) PBACS concentration.**** With fixed TPP (1 mg/mL) and a PBACS:Sil ratio (2:1), PBACS concentrations from 1 to 3.5 mg/mL were tested (Figure S5). Encapsulation efficiency decreased with increasing polymer concentration, potentially due to viscosity-induced diffusion resistance. Particle size showed a non-linear trend, first increasing due to molecular entanglement and later decreasing, possibly due to tighter network formation. A PBACS concentration of 1-2 mg/mL was selected as optimal. ****(D) Stirring time.**** With fixed polymer and drug concentrations, stirring times from 15 to 90 min were evaluated (Figure S5). Longer stirring reduced encapsulation efficiency and drug loading, while particle size increased in the early phase and plateaued after 45 min. PDI remained stable across conditions, with lowest value (0.217) at 15 min, indicating that prolonged stirring may not enhance uniformity. A stirring time of 15–45 min was recommended.

****(E) PBACS:Sil ratio.**** Varying the PBACS:Sil ratio from 4:1 to 1:4 revealed that higher drug concentrations promoted aggregation and turbidity, as reflected in reduced encapsulation efficiency and poor Tyndall effect. Lower drug ratios improved particle size and dispersion quality but reduced drug loading. A PBACS:Sil ratio between 2:1 and 1:2 offered a favorable balance.

**Figure S5.** Effects of different factors on the preparation of Sil@PBACS Nps. (A) TPP concentration (mg/mL); (B) mass ratio of PBACS∶TPP; (C) PBACS concentration (mg/mL); (D) stirring time (min); (E) Mass ratio of PBACS∶Sil. (n = 3)

**2.4 Response surface experiments**

Based on the results from the single-factor experiments, the range of conditions influencing the outcomes was determined. A Plackett-Burman design was then employed using Design-Expert 13 software to optimize the preparation process (Table S1).

**Table S2.** Response Surface Experimental Design

| Factors |  | Level |  |
| --- | --- | --- | --- |
|  | -1 | 0 | 1 |
| TPP Concentration (mg/mL) | 0.5 | 1 | 1.5 |
| PBACS Concentration (mg/mL) | 1 | 1.5 | 2 |
| Time (min) | 15 | 30 | 45 |
| Mass ratio of PBACS:Sil | 1:2 | 1.25:1 | 2:1 |

The results of the response surface experiments for the preparation of nanoparticles are summarized in Table S2. In the models for the four factors, all model terms were significant (*P<*0.05), while the lack-of-fit terms were not significant, indicating that the models were reliable. The adjusted coefficient of determination (R^2^) was 0.955, meaning that 95.5% of the variation in the response values could be explained by the model. Yusuf et al. (2020) have suggested that in studies utilizing response surface methodology, an R^2^ value greater than 0.7 is considered acceptable. Therefore, the response surface model for predicting the Sil-NP preparation process was determined to be feasible and reliable.

The response surface plots in Figure S6 illustrate the interaction effects of various factors on the preparation of Sil nanoparticles. The 3D plots reveal curved surfaces with distinct peaks, representing the optimal conditions for achieving desirable nanoparticle properties. These response surfaces highlight the significant influence of interactions between factors, such as PBACS concentration and TPP concentration, on encapsulation efficiency, drug loading, particle size, and PDI.

Among these, the interaction between PBACS and TPP concentrations exhibited the steepest response curve, indicating the greatest impact on encapsulation efficiency. These insights provide a comprehensive understanding of the combined effects of factors, facilitating the fine-tuning of nanoparticle preparation for optimal performance.

**Figure S6.** Response surface plots showing interaction effects of factors on silymarin nanoparticles**.**

**Table S3. Response Surface Experiments Results**

| Run | TPP Concentration  (mg/mL) | PBACS Concentration (mg/mL) | Time (min) | PBACS:Sil | Encapsulation Efficiency (%) | Drug Loading (%) | Particle Size (nm) | PDI |
| --- | --- | --- | --- | --- | --- | --- | --- | --- |
| 1 | 1.5 | 2 | 30 | 1.25:1 | 48.77 | 6.44 | 412.2 | 0.422 |
| 2 | 1 | 1.5 | 30 | 1.25:1 | 61.47 | 8.11 | 242.5 | 0.344 |
| 3 | 1 | 1 | 15 | 1.25:1 | 71.52 | 9.44 | 182.1 | 0.214 |
| 4 | 1.5 | 1.5 | 45 | 1.25:1 | 69.46 | 9.17 | 356.5 | 0.772 |
| 5 | 1.5 | 1.5 | 15 | 1.25:1 | 61.56 | 8.13 | 232.9 | 0.311 |
| 6 | 1 | 1.5 | 30 | 1.25:1 | 65.56 | 8.65 | 198.7 | 0.176 |
| 7 | 0.5 | 1.5 | 15 | 1.25:1 | 58.30 | 7.70 | 189.7 | 0.143 |
| 8 | 1 | 1.5 | 45 | 2:1 | 59.38 | 5.88 | 171.5 | 0.229 |
| 9 | 1 | 2 | 45 | 1.25:1 | 60.48 | 7.98 | 347.9 | 0.437 |
| 10 | 1 | 1.5 | 45 | 1:2 | 81.75 | 16.19 | 359.1 | 0.359 |
| 11 | 0.5 | 1 | 30 | 1.25:1 | 67.35 | 8.89 | 171.0 | 0.200 |
| 12 | 1 | 2 | 15 | 1.25:1 | 63.40 | 8.37 | 385.2 | 0.724 |
| 13 | 0.5 | 1.5 | 30 | 2:1 | 65.62 | 6.50 | 262.1 | 0.353 |
| 14 | 1.5 | 1.5 | 30 | 1:2 | 78.14 | 15.47 | 265.3 | 0.14 |
| 15 | 0.5 | 2 | 30 | 1.25:1 | 57.72 | 7.62 | 343.2 | 0.392 |
| 16 | 1 | 1 | 30 | 2:1 | 68.70 | 6.80 | 169.6 | 0.255 |
| 17 | 1 | 1.5 | 15 | 2:1 | 57.26 | 5.67 | 177.5 | 0.197 |
| 18 | 1.5 | 1.5 | 30 | 2:1 | 44.43 | 1.43 | 189.1 | 0.280 |
| 19 | 1 | 1 | 45 | 1.25:1 | 65.32 | 8.62 | 159.2 | 0.200 |
| 20 | 1 | 1.5 | 15 | 1:2 | 78.98 | 15.64 | 331.8 | 0.128 |
| 21 | 1 | 2 | 30 | 2:1 | 45.71 | 4.53 | 368.6 | 0.676 |
| 22 | 0.5 | 1.5 | 30 | 1:2 | 81.62 | 16.16 | 238.5 | 0.127 |
| 23 | 1 | 1.5 | 30 | 1.25:1 | 68.83 | 9.08 | 396.0 | 0.555 |
| 24 | 1 | 1.5 | 30 | 1.25:1 | 69.21 | 9.14 | 387.5 | 0.539 |
| 25 | 1.5 | 1 | 30 | 1.25:1 | 63.67 | 8.41 | 179.0 | 0.196 |
| 26 | 1 | 2 | 30 | 1:2 | 76.90 | 15.23 | 431.8 | 0.482 |
| 27 | 1 | 1.5 | 30 | 1.25:1 | 65.43 | 11.28 | 220.3 | 0.158 |
| 28 | 1 | 1 | 30 | 1:2 | 88.48 | 17.52 | 245.9 | 0.307 |
| 29 | 0.5 | 1.5 | 45 | 1.25:1 | 62.33 | 8.23 | 320.9 | 0.544 |

Based on the response surface methodology results presented in Tables S1-S4, the optimal preparation conditions for the nanoparticles were determined to be the following: TPP concentration of 1.25 mg/mL, PBACS concentration of 1 mg/mL, stirring time of 45 minutes, and a drug-to-grafted chitosan ratio of 1:2. Under these conditions, the prepared nanoparticles exhibited an encapsulation efficiency of 86.1 ± 0.2%, a drug loading capacity of 17.4 ± 0.1%, a particle size of 276.3 ± 5.4 nm, and a PDI of 0.228 ± 0.07. These results were closely aligned with the predicted values of encapsulation efficiency (85.5%), drug loading (17.2%), particle size (252.5 nm), and PDI (0.219), confirming the feasibility of using response surface methodology to optimize the nanoparticle preparation process.

**Table S4.** ANOVA for regression model with encapsulation efficiency as response value.

| Source | Sum of Squares | df | Mean Square | *F*-value | *P*-value |  |
| --- | --- | --- | --- | --- | --- | --- |
| Model | 0.267 | 14 | 0.019 | 7.820 | 0.0002 | significant |
| A-TPP concentration | 0.006 | 1 | 0.006 | 2.470 | 0.1385 |  |
| B-PBACS concentration | 0.043 | 1 | 0.043 | 17.700 | 0.0009 |  |
| C-Time | 0.001 | 1 | 0.001 | 0.202 | 0.6596 |  |
| D- PBACS: Sil ratio | 0.174 | 1 | 0.174 | 71.440 | < 0.0001 |  |
| AB | 0.001 | 1 | 0.001 | 0.283 | 0.6027 |  |
| AC | 0.000 | 1 | 0.000 | 0.154 | 0.7006 |  |
| AD | 0.007 | 1 | 0.007 | 3.210 | 0.0951 |  |
| BC | 0.000 | 1 | 0.000 | 0.110 | 0.7449 |  |
| BD | 0.003 | 1 | 0.003 | 1.330 | 0.2680 |  |
| CD | 0.000 | 1 | 0.000 | 0.004 | 0.9483 |  |
| A² | 0.009 | 1 | 0.009 | 4.000 | 0.0654 |  |
| B² | 0.001 | 1 | 0.001 | 0.596 | 0.4529 |  |
| C² | 0.000 | 1 | 0.000 | 0.000 | 0.9860 |  |
| D² | 0.013 | 1 | 0.013 | 5.660 | 0.0321 |  |
| Residual | 0.034 | 14 | 0.002 |  |  |  |
| Lack of fFit | 0.030 | 10 | 0.003 | 3.090 | 0.1443 | not significant |
| Pure error | 0.003 | 4 | 0.001 |  |  |  |
| Cor total | 0.302 | 28 |  |  |  |  |

**Table S5.** ANOVA for regression model with drug loading as response value.

| Source | Sum of Squares | df | Mean Square | *F*-value | *P*-value |  |
| --- | --- | --- | --- | --- | --- | --- |
| Model | 0.040 | 14 | 0.002 | 24.730 | < 0.0001 | significant |
| A-TPP concentration | 0.000 | 1 | 0.000 | 2.580 | 0.130 |  |
| B-PBACS concentration | 0.000 | 1 | 0.000 | 6.390 | 0.024 |  |
| C-Time | 0.000 | 1 | 0.000 | 0.090 | 0.768 |  |
| D- PBACS: Sil ratio | 0.035 | 1 | 0.035 | 302.180 | < 0.0001 |  |
| AB | 0.000 | 1 | 0.000 | 0.100 | 0.754 |  |
| AC | 0.000 | 1 | 0.000 | 0.050 | 0.817 |  |
| AD | 0.000 | 1 | 0.000 | 4.060 | 0.063 |  |
| BC | 0.000 | 1 | 0.000 | 0.030 | 0.845 |  |
| BD | 7.330 | 1 | 0.000 | 0.000 | 0.994 |  |
| CD | 0.000 | 1 | 0.000 | 0.020 | 0.878 |  |
| A² | 0.000 | 1 | 0.000 | 5.790 | 0.030 |  |
| B² | 0.000 | 1 | 0.000 | 0.530 | 0.475 |  |
| C² | 0.000 | 1 | 0.000 | 0.150 | 0.701 |  |
| D² | 0.002 | 1 | 0.002 | 18.460 | 0.001 |  |
| Residual | 0.001 | 14 | 0.000 |  |  |  |
| Lack of fit | 0.001 | 10 | 0.000 | 0.740 | 0.682 | not significant |
| Pure error | 0.000 | 4 | 0.000 |  |  |  |
| Cor total | 0.042 | 28 |  |  |  |  |

**Table S6.** ANOVA for regression model with particle size as response value.

| Source | Sum of Squares | df | Mean Square | F-value | *P*-value |  |
| --- | --- | --- | --- | --- | --- | --- |
| Source | 148900.000 | 10 | 14890.180 | 3.630 | 0.0085 | significant |
| Model | 1001.010 | 1 | 1001.010 | 0.244 | 0.6272 |  |
| A-TPP Concentration | 116400.000 | 1 | 116400.000 | 28.400 | < 0.0001 |  |
| B-PBACS Concentration | 3884.400 | 1 | 3884.400 | 0.947 | 0.3433 |  |
| C-Time | 23763.000 | 1 | 23763.000 | 5.800 | 0.027 |  |
| D- PBACS: Sil ratio | 930.250 | 1 | 930.250 | 0.227 | 0.6396 |  |
| AB | 14.440 | 1 | 14.440 | 0.004 | 0.9533 |  |
| AC | 2490.010 | 1 | 2490.010 | 0.607 | 0.4459 |  |
| AD | 51.840 | 1 | 51.840 | 0.013 | 0.9117 |  |
| BC | 42.900 | 1 | 42.900 | 0.011 | 0.9197 |  |
| BD | 277.220 | 1 | 277.220 | 0.068 | 0.7978 |  |
| CD | 73807.390 | 18 | 4100.410 |  |  |  |
| Residual | 37620.110 | 14 | 2687.150 | 0.297 | 0.9604 | not significant |
| Lack of Fit | 36187.280 | 4 | 9046.820 |  |  |  |
| Pure Error | 222700.000 | 28 |  |  |  |  |
| Cor Total |  |  |  |  |  |  |

**Table S7.** ANOVA for regression model with polydispersity index as response value.

| Source | Sum of Squares | df | Mean Square | F-value | P-value |  |
| --- | --- | --- | --- | --- | --- | --- |
| Model | 0.342 | 4 | 0.0856 | 3.305 | 0.0271 | significant |
| A-TPP Concentration | 0.010 | 1 | 0.010 | 0.421 | 0.5223 |  |
| B-PBACS Concentration | 0.258 | 1 | 0.258 | 9.973 | 0.0042 |  |
| C-Time | 0.056 | 1 | 0.056 | 2.183 | 0.1524 |  |
| D- PBACS: Sil ratio | 0.016 | 1 | 0.016 | 0.642 | 0.4306 |  |
| Residual | 0.621 | 24 | 0.025 |  |  |  |
| Lack of Fit | 0.477 | 20 | 0.023 | 0.658 | 0.7650 | not significant |
| Pure Error | 0.144 | 4 | 0.036 |  |  |  |
| Cor Total | 0.964 | 28 |  |  |  |  |

**Table S8.** Comparison of the stability of drug-loaded nanoparticles and blank nanoparticles (n = 3).

| Group | Particle Size (nm) | PDI | Zeta potential (mV) |
| --- | --- | --- | --- |
| PBACS Nps | 201.3±7.1^C^ | 0.491±0.05^C^ | 34.4±1.7^a^ |
| PBACS@Sil Nps | 276.3±6.3^B^ | 0.228±0.07^B^ | 31.8±1.2^a^ |
| PBACS@Sil Nps (1 w) | 279.3±6.1^B^ | 0.214±0.017^B^ | 32.5±1.9^a^ |
| PBACS@Sil Nps (1 M) | 281.4±5.7^B^ | 0.221±0.011^B^ | 31.4±2.1^a^ |
| PBACS@Sil Nps (3 M) | 280.2±8.2^B^ | 0.197±0.019^B^ | 31.2±2.7^a^ |
| PBACS@Sil Nps (6 M) | 718.7±7.6^a^ | 0.542±0.017^a^ | 28.9±1.8^a^ |

Data in the same column labeled with the same letter indicates nonsignificant difference (*P* > 0.05); different letters indicate a significant difference (*P*< 0.05); and capital letters indicate a highly significant difference (*P*< 0.01).

**Figure S7.** The random forest model screened out significant expression biomarkers with MDA values >4

**Figure S8.** Permutation test for the OPLS-DA model.

Permutation test for the OPLS-DA model. The x-axis represents the correlation coefficient between the permuted and original class labels. The y-axis represents the values of R²Y and Q². The green and blue dots represent the R²Y and Q² values, respectively, of the models built with randomly permuted data. The solid/dashed lines are the regression lines for R²Y and Q². The intercepts of the regression lines are R² = (0.0, 0.92) and Q² = (0.0, -0.4), with the Q² intercept being less than zero, indicating that the model is valid and not overfitted. The number of permutation tests was 200.

**Table S9.** Differential metabolites between the nanoparticle-treated group and the model group (*p* < 0.01).

| id | MS2 name | MS2 score | mz | rt | MEAN cos-7 | MEAN mod-7 | VIP | P-VALUE | Q-VALUE | FOLD CHANGE | LOG_FOLDCHANGE |
| --- | --- | --- | --- | --- | --- | --- | --- | --- | --- | --- | --- |
| 498 | Creatinine | 0.9997 | 114.065873 | 161.18 | 3.758890113 | 39.45655609 | 2.192179349 | 0.003648054 | 0.256942624 | 0.095266554 | -3.391886392 |
| 648 | Phenylethylamine | 0.8883 | 122.0951976 | 163.646 | 0.244538696 | 0.572890065 | 2.127843992 | 0.008359893 | 0.298129982 | 0.426850997 | -1.228195546 |
| 1270 | trans-cinnamate | 0.7677 | 149.0582962 | 239.873 | 0.026419173 | 0.087568006 | 2.14468083 | 0.008391934 | 0.298271319 | 0.301698919 | -1.728818568 |
| 1291 | D-Ribose | 0.35355 | 150.0529275 | 101.072 | 0.368729582 | 0.817927139 | 2.144061635 | 0.000524589 | 0.198001407 | 0.450809814 | -1.149409171 |
| 1341 | 2-Hydroxyadenine | 0.8838 | 152.0551712 | 214.225 | 0.068643942 | 0.351780975 | 2.116423203 | 0.006572211 | 0.288394356 | 0.195132616 | -2.357473152 |
| 1377 | 3-Hydroxyanthranilic acid | 0.4866 | 154.0491213 | 328.542 | 2.931276448 | 1.749709418 | 1.52112564 | 0.006418125 | 0.287340883 | 1.67529329 | 0.744413687 |
| 1391 | Glycerol 3-phosphate | 0.9416 | 155.008914 | 43.201 | 0.110014414 | 0.281718753 | 1.823128844 | 0.005632607 | 0.281230177 | 0.390511504 | -1.356563046 |
| 1570 | 1-Aminocyclopropanecarboxylic acid | 0.5 | 162.073887 | 456.9915 | 0.505104874 | 0.212410593 | 1.369124905 | 0.009089279 | 0.301129003 | 2.377964615 | 1.249727247 |
| 1594 | 2-Hydroxy-3-methylbutyric acid | 0.37415 | 163.0382502 | 40.636 | 0.057468572 | 0.087421244 | 1.688795764 | 0.00990732 | 0.304023295 | 0.657375359 | -0.605210714 |
| 1659 | L-Phenylalanine | 0.9963 | 166.0847981 | 240.413 | 0.258123423 | 2.151113944 | 2.148320923 | 0.007536575 | 0.294142037 | 0.119995235 | -3.058950979 |
| 1726 | Norharmane | 0.7485 | 169.0743094 | 44.098 | 0.122605019 | 0.328413704 | 1.630298235 | 0.004473598 | 0.26911543 | 0.373324918 | -1.42149629 |
| 3053 | Val-Val | 0.9903 | 217.1536844 | 201.261 | 0.053795248 | 0.174318293 | 2.035714628 | 0.005173551 | 0.276960062 | 0.308603573 | -1.696173328 |
| 3319 | Formylanthranilic acid | 0.4092 | 226.0692262 | 289.463 | 14.78996288 | 8.983775364 | 1.725616502 | 0.009871642 | 0.303905994 | 1.64629705 | 0.719224672 |
| 3391 | Pyridoxal (Vitamin B6) | 0.38255 | 228.0850112 | 42.796 | 0.262661924 | 1.032011893 | 2.135845607 | 0.007035046 | 0.291321205 | 0.254514435 | -1.974180614 |
| 3591 | Nalidixic acid | 0.46225 | 233.089607 | 161.0775 | 0.707429808 | 0.09553974 | 2.122425319 | 0.000907919 | 0.198001407 | 7.404560724 | 2.88841415 |
| 3858 | 3-Deoxy-2-keto-6-phosphogluconic acid | 0.3983 | 241.1154506 | 151.395 | 0.34695969 | 0.166677096 | 1.643874042 | 0.004289719 | 0.266721914 | 2.081627881 | 1.05771219 |
| 4029 | Ile-Ile | 0.9996 | 245.1853489 | 170.215 | 0.181745555 | 0.449914001 | 1.923918965 | 0.00848293 | 0.298667606 | 0.40395621 | -1.307729186 |
| 4031 | Leu-Leu | 0.49935 | 245.1863269 | 37.031 | 0.244987437 | 0.490049341 | 2.079392464 | 0.004881517 | 0.273907214 | 0.499924021 | -1.000219247 |
| 4364 | para-Phenylenediamine | 0.3222 | 255.0964257 | 169.5355 | 0.654316329 | 0.076520943 | 2.110871477 | 0.004355919 | 0.26760193 | 8.550813728 | 3.096061719 |
| 4626 | D-Biotin | 0.4762 | 262.1264124 | 378.097 | 0.216759077 | 0.441096509 | 2.063191767 | 0.001128946 | 0.198001407 | 0.49140964 | -1.025001937 |
| 4885 | Tyr-Ser | 0.4871 | 269.1099713 | 161.523 | 45.36108092 | 1.050295126 | 2.255909968 | 0.000967161 | 0.198001407 | 43.18889025 | 5.432588341 |
| 5981 | 3-ketosphinganine | 0.9819 | 300.2897174 | 41.1485 | 54.28374243 | 22.91483406 | 1.760411563 | 0.001308921 | 0.198001407 | 2.368934564 | 1.244238348 |
| 6386 | Heptadecanoic acid | 0.31095 | 312.2875203 | 40.542 | 0.36027128 | 0.227101994 | 1.423499782 | 0.005431048 | 0.279428266 | 1.586385366 | 0.665743275 |
| 8311 | Pregnenolone | 0.36875 | 377.2624888 | 32.792 | 1.557054263 | 0.764598704 | 1.757178063 | 0.000670928 | 0.198001407 | 2.0364333 | 1.026044562 |

This table lists the 24 differential metabolites identified between the nanoparticle-treated and model groups, which were selected as potential biomarkers based on statistical significance (*p* < 0.01).

**Table S10.** Molecular docking binding energies of silymarin with different target proteins.

| Receptor Original name | Gene name | Ligand | Binding energy(kcal/mol) |
| --- | --- | --- | --- |
| E2R5G6 | RRAS | Silymarin | -10.25 |
| J9P6J8 | GBA2 | Silymarin | -10.16 |
| F1Q3W8 | SDHB | Silymarin | -10.11 |
| J9P0Z3 | NDUFA8 | Silymarin | -10.06 |
| F1PIL4 | DYRK1A | Silymarin | -9.946 |
| E2RME9 | SGPL1 | Silymarin | -9.941 |
| F1Q054 | TSC2 | Silymarin | -9.786 |
| F1Q018 | SMPD1 | Silymarin | -9.775 |
| F1P9Z4 | RRAS2 | Silymarin | -9.732 |
| F1P8R0 | STEAP3 | Silymarin | -9.635 |
| A0A5F4C504 | MAP2K4 | Silymarin | -9.595 |
| E2QX84 | HSPA2 | Silymarin | -9.426 |
| F1Q2F | FASN | Silymarin | -9.342 |
| J9P6J8 | GBA2 | Silymarin | -9.238 |
| A0A5F4C504 | MAP2K4 | Silymarin | -9.144 |
| A0A5F4D9C2 | MAP2K3 | Silymarin | -9.029 |
| A0A5F4BYP6 | ACACA | Silymarin | -8.182 |
| E2REC1 | SERPINB5 | Silymarin | -7.766 |
| Q6F3J0 | NFKB1 | Silymarin | -7.603 |
| A0A5F4DFM8 | PPM1A | Silymarin | -7.595 |
| Q8HYU5 | BAX | Silymarin | -7.364 |

This table summarizes the predicted binding energies (kcal/mol) of silymarin with selected target proteins, reflecting the relative binding affinity of each drug–protein complex.

**Table S11.** Primary antibodies used for Western blot.

| Tissue | Target protein | Catalog no. | Supplier | Working dilution |
| --- | --- | --- | --- | --- |
| Murine intestine | ZO-1 | AB2035 | Beyotime | 1:1000 |
|  | Occludin | AF7644 | Beyotime | 1:1000 |
|  | Claudin-1 | AF6504 | Beyotime | 1:1000 |
|  | MUC2 | AG2688 | Beyotime | 1:1000 |
|  | FGF15 | PA5-117645 | Invitrogen | 1:1000 |
|  | TLR4 | AB2062 | Beyotime | 1:1000 |
|  | Phospho-NF-κB p65 (Ser536) | AB3013 | Beyotime | 1:1000 |
|  | NF-κB p65 | AB2020 | Beyotime | 1:1000 |
|  | GAPDH | AF0006 | Beyotime | 1:1000 |
|  | TLR4 | AB2062 | Beyotime | 1:1000 |
| Murine liver | Phospho-NF-κB p65 (Ser536) | AB3013 | Beyotime | 1:1000 |
|  | NF-κB p65 | AB2020 | Beyotime | 1:1000 |
|  | FGFR4 | MA5-42915 | Invitrogen | 1:1000 |
|  | β-Klotho/KLB | PA5-119246 | Invitrogen | 1:1000 |
|  | CYP7A1 | AF6657 | Beyotime | 1:1000 |
|  | PCNA | AF0261 | Beyotime | 1:1000 |
|  | Cyclin D1 | AF0126 | Beyotime | 1:1000 |
|  | BAX | AF5120 | Beyotime | 1:1000 |
|  | Bcl-2 | AB112 | Beyotime | 1:1000 |
|  | ACACA | AF6123 | Beyotime | 1:1000 |
|  | FASN | AF5168 | Beyotime | 1:1000 |
|  | Caspase-3 | AF5132 | Beyotime | 1:1000 |
|  | Cleaved Caspase-3 (Asp175) | AC033 | Beyotime | 1:1000 |
|  | PI3K p85 | AG2874 | Beyotime | 1:1000 |
|  | Akt | AA326 | Beyotime | 1:1000 |
|  | Nrf2 | AF7623 | Beyotime | 1:1000 |
|  | HO-1 | AF1333 | Beyotime | 1:1000 |
|  | NQO1 | AF7614 | Beyotime | 1:1000 |
|  | GAPDH | AF0006 | Beyotime | 1:1000 |

Primary antibodies were incubated overnight at 4 °C. Beyotime Biotechnology, Shanghai, China; Invitrogen, Thermo Fisher Scientific, USA.

This table summarizes the primary antibodies used for Western blot, including the tissue, target protein, catalogue number, supplier, and working dilution.
